# Processing-Induced Changes in Phenolic Composition and Dough Properties of Grape Pomace-Enriched Wheat Buns

**DOI:** 10.3390/foods14244256

**Published:** 2025-12-10

**Authors:** Václav Dvořáček, Michal Jágr, Michael Jelínek, Lucie Jurkaninová, Adéla Fraňková

**Affiliations:** 1 Czech Agrifood Research Center, Drnovská 507, 161 06 Prague, Czech Republic; michal.jagr@carc.cz (M.J.); michael.jelinek@carc.cz (M.J.); 2 Department of Food Science, Faculty of Agrobiology, Food and Natural Resources, Czech University of Life Sciences, Kamýcká 129, 165 00 Praha, Czech Republic; frankovaa@af.czu.cz

**Keywords:** grape pomace, phenolic compounds, anthocyanins, bakery processing, rheology, LC-MS, Mixolab, FTIR

## Abstract

The study aimed to elucidate compositional changes in free phenolic compounds (fPHEs) during bakery processing of wheat flour supplemented with grape pomace (GP) and to assess dough rheology, bun shape and physical characteristics. Three GP variants were used—two from white cultivars (Rhine Riesling; Rhine Riesling + Muscat of Moravia) and one from a red blend (Saint Laurent and André)—at substitution levels of 5, 10, 20, and 30%. Thirty-four fPHEs were quantified by high-resolution UHPLC-MS-Orbitrap; dough rheology was assessed by Mixolab; and potential fPHE–wheat macromolecule interactions were examined via FTIR spectroscopy. Wheat flour contained only six fPHEs at low concentrations. Both white GP samples had similar profiles of 32 fPHEs, dominated by miquelianin (526–683 µg/g) and hyperoside + isoquercetin (390–476 µg/g). Red GP was highly enriched in anthocyanins (>30,000 µg/g) and generally exceeded white GP in most fPHEs. Even 5% GP substantially increased fPHE concentrations throughout processing. Several compounds (e.g., gallic acid, miquelianin) exceeded theoretical values, suggesting release from bound forms during fermentation and heating, whereas anthocyanins lost at least 30% during baking. Rheological analysis showed shorter dough development and reduced stability with increasing GP. White GP enhanced starch gelatinization (C3), gel stability (C4), and retrogradation, whereas 20% red GP markedly impaired gelatinization. GP additions ≥10% deteriorated bun shape and physical properties. FTIR confirmed spectral shifts likely due to fPHE–protein/starch interactions. In summary, incorporation of just 5% GP enhanced the nutritional profile of wheat buns.

## 1. Introduction

The wine industry generates substantial waste, particularly grape pomace, discarded grapes, seeds, and sediments, with pomace accounting for approximately 30% of the original grape weight. Most wineries utilize it as animal feed, compost, or dispose of the pomace without prior treatment, which can lead to soil contamination and acidification [1]. However, grape pomace is rich in phenolic compounds (PHEs), dietary fiber, proteins, and minerals, making it a promising ingredient for fortifying food products, especially cereal-based items such as bread, cookies, and pasta. The main bioactive constituents of grape pomace include anthocyanins, hydroxycinnamic acids, catechins, and flavonols, which can inhibit cellular oxidative processes and may contribute to the prevention of cardiovascular diseases, cancer, osteoporosis, and neurodegenerative disorders [2,3,4].

Frequently analyzed groups of PHEs in grape pomace include flavan-3-ols, anthocyanins, flavonols, and non-flavonoids such as phenolic acids (PHAs) and stilbenes. Their concentrations vary widely—from tens of µg/g dry matter for stilbenes, through hundreds of µg/g for PHAs, to several thousand µg/g for anthocyanins. This variability depends on grape cultivar, origin, and winemaking processes; even within a single cultivar, differences may occur due to geographic region and vintage [5,6]. Compared to grape pomace, wheat flour—both refined and wholegrain—contains substantially lower levels of free flavonoids and PHAs, typically only a few µg/g dry matter [7].

The health effects of dietary polyphenols largely depend on their bioavailability, defined as the fraction of polyphenols released from the food matrix, metabolized, absorbed, and capable of exerting biological activity in target cells or tissues. Factors affecting bioavailability include their initial content in foods, the food matrix, gut microbiota composition, and food processing methods such as heating, drying, or fermentation [8].

Skrajda-Brdak et al. [9] reported that in cereals only a small fraction of PHAs is readily available from the digestive tract, as the majority (up to 99%) exists as esters, ethers, and amides bound to arabinoxylans, forming a lignocellulosic matrix resistant to digestion. Enrichment of bakery products with dried grape pomace, rich in free phenolic acids (fPHAs), even at low substitution levels in flour (5–7%), can substantially increase their bioavailable content in the final product.

The bioavailability of polyphenols is also sensitive to processing conditions such as temperature, exposure time, moisture, oxygen, pH, molecular transformations, and metabolism. Moreover, PHEs may form complexes with starch, proteins, and polysaccharides, reducing their bioavailability [10]. Conversely, current strategies aimed at enhancing the bioavailability of heat-, light-, and pH-sensitive free anthocyanins exploit their interactions with polysaccharides or proteins, thereby preserving their stability and improving their bioactivity [11].

The potential to enhance the nutritional value of bakery and pastry products (e.g., muffins, cookies, wafers, cakes, or buns) through the addition of grape pomace has been investigated in several studies. Results indicate that even a low incorporation level of grape pomace (5–10%) in wheat flour can significantly increase PHE content, dietary fiber, and antioxidant capacity of the final products without markedly affecting sensory or technological properties [2,12].

In view of these findings, our study focused on detailed monitoring of compositional changes in a broad spectrum of free phenolic compounds (fPHE) (34 detected compounds) in wheat blends enriched with different types and levels of grape pomace during successive stages of bakery processing. We also analyzed the rheological behavior of these blends, with particular attention to modifications in the starch–amylase complex depending on increasing phenolic content and their potential interactions with wheat starch. Available information on these processes remains limited, and a deeper understanding of changes in fPHEs from grape pomace during baking could contribute not only to higher bioavailability in the final product but also to alleviating the negative impact of grape pomace on the texture and structural quality of baked products.

For the analysis, we employed advanced techniques, namely UHPLC-MS-Orbitrap for precise quantification of PHEs, the Mixolab I system for comprehensive evaluation of the rheological properties of the blends, and FT-IR spectroscopy to monitor potential interactions between PHEs and wheat starch. This integrated approach offered an enhanced understanding of the relationships between PHEs and wheat starch during bakery processing.

## 2. Materials and Methods

### 2.1. Material

Three distinct samples of dried grape pomace (approximately 10 kg per sample) were obtained from the private enterprise Ludwig Winery Ltd. (Roztoky u Prahy, South Moravia, Czech Republic) for subsequent evaluation. Sample No. 1 consisted exclusively of the white variety Rhine Riesling (RR). Sample No. 2 was a 1:1 (*w*/*w*) mixture of the white varieties Rhine Riesling and Muscat of Moravia (RR + MM). Sample No. 3 was a 1:1 (*w*/*w*) blend of the red grape varieties Saint Laurent and André (RED). These three grape pomace samples were selected as model representatives of typical white and red pomaces, capturing key differences in phenolic composition and technological behavior. The two white samples allowed assessment of variability within white cultivars, while the red sample provided a phenolic- and anthocyanin-rich counterpart for comparison. Immediately after pressing, the grape pomace was transferred to a prototype belt drying line equipped with microwave heating, designed and constructed by Prokop Technology Ltd. (Mnichovice–Božkov, Czech Republic) and installed at the Ludwig Winery. The dryer consisted of six sections with 35 magnetrons each (total 210, type 2M248 K, 1030 W, 2460 Mhz) cooled centrally by liquid. Continuous drying was performed for approximately 30–35 min per 20 m of belt travel, until final moisture content of 10–11% was reached. The magnetron power in each section was regulated based on reaching a maximum local temperature of 60 °C. After drying, the material was milled into a fine powder using an industrial impact mill positioned downstream of the drying unit (Prokop Technology, Czech Republic).

The resulting powdered samples, together with a commercial fine wheat flour (sourced from Perner Mill, Svijany, Czech Republic), were subjected to chemical analyses with a particular focus on the quantification of selected PHEs. These raw materials were subsequently used for the preparation of wheat buns with defined levels of grape pomace substitution. Additional analyses addressed the variability of PHEs throughout the processing phases, combined with rheological characterization of the dough and screening evaluation of the technological and sensory quality of the final buns.

### 2.2. Basic Chemical Analyses of Grape Pomace

Basic chemical analyses of the obtained grape pomaces included the determination of crude protein, crude fiber, fat, glucose, and fructose. Mean values from three independent measurements are summarized in Supplementary Table S1.

Crude protein was determined by the Kjeldahl method (ČSN EN ISO 5983-1 [13]). Fat content was analyzed using the Soxterm 2000 system (Gerhardt GmbH & Co., Königswinter, Germany) based on Soxhlet-Twisselmann extraction AOAC Method 920.39 [14]. Glucose and fructose were quantified by RP-HPLC (Waters 2965 system; Waters Corporation, Milford, MA, USA) with a LiChrospher® NH2 column (250 mm × 4 mm, 5 μm; Merck KGaA, Darmstadt, Germany) and refractometric detection [15]. Crude fiber was determined using the ANKOM DELTA Fiber Analyzer (ANKOM Technology, Macedon, NY, USA) according to AOCS Official Method Ba 6a-05. Ash content was determined by incineration of the samples at 550 °C until constant weight, according to AOAC Official Method 923.03.

### 2.3. Rheological Analysis of Wheat Flour Substituted with Grape Pomace

Selected combinations of control flour and flours with defined levels of grape pomace substitution (5, 10, 20 and 30%) were rheologically evaluated using the Mixolab I analyzer (Chopin Technologies, Villeneuve la-Garenne, France) according to ICC N°173 [16]. The protocol for the analysis of isolated starch was derived from the standard “Chopin+.” The standard Mixolab curve is generally characterized by five points (C1–C5). C1 is the time for achievement of optimal torque (1.1 Nm) of dough during mixing at 30 °C; C2 represents protein weakening during temperature increase and dough stability (DS); C3 denotes starch gelatinization; C4 describes the stability of the hot-formed gel; and C5 indicates starch retrogradation during the cooling period. The rheological curve analysis also provides information about point differences: C3–C2 (gelatinization intensity), C3–C4 (intensity of amylase activity), and C5–C4 (extent of retrogradation) [17].

### 2.4. Analysis of the Phenolic Compounds

#### 2.4.1. Reference Standards for Phenolic Compounds and Reagents

Commercially available phenolic standards (36 compounds in total) were used for quantitative analyses, along with two internal standards for calibration purposes. Phenolic standards from Sigma Aldrich (St. Louis, MO, USA) included astilbin, caffeic acid, catechin, chlorogenic acid, ferulic acid, gallic acid, p-hydroxybenzoic acid, p-coumaric acid, hyperoside, isoquercetin, kaempferol, miquelianin, myricetin, quercetin, quercitrin, rutin, syringic acid, taxifolin, trifolin, and vanillic acid, as well as the internal standards verapamil hydrochloride and probenecid. Standards obtained from Toronto Research Chemicals Inc. (Toronto, Canada; www.lgcstandards.com) were cis-resveratrol, trans-resveratrol, epicatechin, and neochlorogenic acid. Phenolic standards purchased from ChemFaces (Wuhan, China; www.chemfaces.com) included catechin gallate, delphinidin-3-O-galactoside, epicatechin gallate, epigallocatechin, epigallocatechin gallate, gallocatechin, gallocatechin gallate, malvidin-3-O-galactoside, petunidin-3-O-glucoside, 13 procyanidin A2, procyanidin B1 + B3 and procyanidin B2.

Methanol (LC-MS grade, ≥99.9%) was obtained from Riedel de Haën (Seelze, Germany). Formic acid (LC-MS grade, 99%) was purchased from VWR (Leuven, Belgium). Pure water was obtained from a Milli-Q purification system (Millipore, Bedford, MA, USA).

#### 2.4.2. Phenolic and Internal Standards Preparation and Sample Extraction

To prepare reference stock solutions, reference standards of individual PHEs and internal standards were dissolved in methanol to obtain a stock solution of 0.5 mg/mL, which were stored at −18 °C. The stocks were further diluted with methanol to 1−10,000 ng/mL to create calibration curves for PHEs quantification. Verapamil and probenecid were dissolved in methanol at 0.5 mg/mL to prepare a stock solution of the internal standards. Verapamil and probenecid were then added into PHEs standard solutions or test samples to the final concentration of 100 ng/mL.

The PHE extraction procedure was based on a procedure taken from our previous study [18]. Briefly, 0.1 g of the sample was extracted twice with 1 mL of extraction solvent (80% methanol containing probenecid and verapamil as internal standards at a concentration of 0.1 g/mL) in Eppendorf tubes for 60 min at 45 °C using an ultrasonic bath. The samples were then centrifuged for 10 min at 13,500 rpm. The resulting supernatants from each sample were filtered through 0.2 μm nylon syringe filters (Thermo Scientific, Rockwood, TN, USA). Extracts were prepared no more than 2 days prior to UHPLC-ESI-MS/MS analysis and stored at −18 °C. Phenolic compounds were extracted from both the prepared dough samples and the final baked bun samples. For the final bun, the entire sample was finely ground to obtain a homogeneous material prior to extraction of phenolic compounds.

Three independent sample collections (biological replicates) were performed, and each sample was analyzed in triplicate (technical replicates) to ensure reproducibility and reliability of the measurements.

#### 2.4.3. UHPLC-ESI-MS/MS Instrumentation and Analysis

LC-MS/MS analyses were performed on a chromatographic system (Dionex UltiMate 3000 UHPLC system, Dionex Softron GmbH, Germering, Germany) which consisted of a binary pump (HPG-3400RS), an autosampler (WPS-3000RS), a degasser (SRD-3400), and a column oven (TCC-3000RS). Detection was performed on a quadrupole/orbital ion trap Q Exactive mass spectrometer (Thermo Fisher Scientific, San Jose, CA, USA). Analytes were separated on a Acquity Premier HSS T3 column (2.1 × 100 mm, 1.8 µm) from Waters (www.waters.com). Chromatographic separation was performed using gradient elution with 0.1% formic acid (*v*/*v*) in water as a solvent A, and methanol with 0.2% formic acid (*v*/*v*) as solvent B. The LC gradient started at 0 min with 0% of solvent B, then linear gradient to 60% B in 11.0 min was applied. The column was flushed with 100% of B in 13 min and then column was equilibrated back to 0% B in 15 min. The column was maintained at 40 °C at a flow rate of 0.35 mL/min.

The mass spectrometer was generally operated in a positive or negative electrospray ionization (ESI+/ESI−) mode with a high-resolution accurate-mass full scan setting with 70,000 FWHM resolution. For quantitative analysis of target compounds, a parallel reaction monitoring (PRM) experiment was used with 17,500 FWHM for MS/MS scans. Normalized collision energy (NCE) for individual PHEs was set individually (Table 1). Spray voltage was maintained at 3.5 kV in the positive ion mode and at −2.5 kV in the negative ion mode. Sheath gas flow was 49 arbitrary units, auxiliary gas flow rate was kept at 12 arbitrary units, and sweep gas flow was 2 arbitrary units. Capillary temperature was 259 °C. Nitrogen was used as sheath, auxiliary, and sweep gas. Heater temperature was kept at 419 °C. S-lens RF level was 50 for positive ion mode and 30 for negative ion mode. The accuracy and calibration of the Q Exactive Orbitrap LC-MS/MS was checked using a reference standard mixture (Pierce LTQ Velos ESI Positive or Negative Ion Calibration Solution) obtained from Thermo Fisher Scientific. Data were evaluated by the Quan/Qual Browser Xcalibur software, v 4.0.

### 2.5. Wheat Bun Preparation, Sampling, Shape and Physical Analyses

Wheat flour was partially substituted with three types of grape pomace at levels of 5, 10, 20, and 30% (*w*/*w*). A control sample without pomace was also prepared. The basic formulation consisted of 300 g of wheat flour or a mixture of wheat flour and grape pomace, 12 g of yeast, 3 g of fat (a commercial butter), 4.5 g of sugar, 5.1 g of salt, 1.5 g of bread improver (commercial diastatic malt flour), and water according to the farinograph absorption. In the experimental variants, part of the flour was replaced by the corresponding proportion of pomace.

Doughs were prepared in a farinograph at 30 °C and adjusted to 550–650 farinograph units (FU). They underwent two fermentation stages (45 min and 50 min) at 30 °C and 85% relative humidity in a fermentation chamber. The fermented doughs were divided after the first fermentation stage into 80 g portions, shaped into buns, and baked at 240 °C for 14 min with an initial steam injection of 70 mL of distilled water. For each variant, buns were prepared in triplicate, cooled for 90 min, and subsequently evaluated.

The geometric characteristics of the baked buns (height, width, and height-to-width ratio) were assessed. The average volume of three buns per variant was determined using the volumetric displacement method with rapeseed (*Brassica napus*) seeds. To express volume-related parameters, two metrics were calculated: bread yield/100 g flour = volume of the buns per 100 g of flour or flour–grape pomace mixture (cm^3^/100 g of wheat flour or mixture), and specific volume following the AACCI Approved Method 10-05.01 (AACC-I, 2009 [19]), defined as the volume per 100 g of baked product (cm^3^/100 g of bun).

Samples were collected at four stages: (i) dough before fermentation, (ii) dough after the first fermentation, (iii) dough after the second fermentation, and (iv) bun after baking. Prior to lyophilization and PHE analysis, approximately 10 g of each sample was taken across the central section of the bun, including both crumb and crust, lyophilized (Christ Beta 1–8 LD plus) for 48 h at −50 °C under vacuum, and analyzed for PHE content. In parallel, about 250 g of each substituted flour variant (including the control without pomace) was allocated for rheological analysis using a Mixolab I device (see Section 2.5).

### 2.6. Fourier Transform Infrared (FTIR) Analysis

Lyophilized and finely ground samples (Perten Mill 3300) of baked wheat buns, including the control bun and buns with 30% substitution of both white and red grape pomace, were analyzed, and spectral variations were compared. FTIR spectra were acquired using a Nicolet^TM^ IS50 FT-IR Spectrometer (Thermo Fisher Scientific, Madison, WI, USA) with OMNIC software (Version 9.0, Thermo Fisher Scientific, Madison, WI, USA) over a wavelength range of 400–4000 cm^−1^. Powdered samples were placed in contact with the attenuated total reflectance (ATR) diamond crystal at 25 °C. Each spectrum was collected as an average of 32 scans in two independent measurements, and results were reported as mean values. Spectra were ATR-corrected, smoothed, and baseline-corrected using OMNIC software prior to the generation of output graphs.

### 2.7. Statistical Analysis

Relative changes in fPHEs during dough preparation, fermentation, and baking were evaluated using two parameters: the theoretical pomace ratio (*TPR*) and the equivalent pomace ratio (*EPR*). For each phenolic compound *i* and each sample variant *k*—defined as the specific combination of processing stage and pomace level—the following procedure was applied.

The theoretical pomace ratio *TPRₖ* represents the defined proportion of grape pomace in the flour mixture (5, 10, 20, or 30%). Assuming no degradation, release from bound forms, or matrix interactions, the theoretical concentration of phenolic compound *i* in the mixture is given by

$$C_{i,k}^{theor}=TPR_{k}\cdot C_{i}^{pom},$$

where $C_{i}^{pom}$ is the concentration of phenolic compound *i* in pure grape pomace.

The equivalent pomace ratio *EPRᵢ*_,_*ₖ* expresses the pomace proportion that would be required to achieve the *measured* concentration $C_{i,k}^{meas}$ of phenolic compound *i* in sample variant *k*. It is calculated as

$$EPR_{i,k}=TPR_{k}\cdot \frac{C_{i,k}^{meas}}{C_{i,k}^{theor}}.$$

This transformation normalizes the data across phenolic compounds with highly variable baseline concentrations, enabling direct comparison of relative trends during processing.

Values of EPR larger than TPR indicate a relative increase in the phenolic compound beyond theoretical expectations (e.g., release from bound forms or improved extractability), whereas EPR values lower than TPR indicate a relative decrease (e.g., degradation, binding, or dilution effects).

Data were statistically evaluated using Statistica software (Version 7.1; StatSoft, Inc., Tulsa, OK, USA). The analysis included the calculation of basic descriptive statistics (mean, minimum, and maximum), Spearman’s correlation coefficients, factorial analysis of variance (ANOVA), Tukey’s HSD post hoc test, and graphical plotting.

Since the measured changes in PHEs during bun processing markedly violated the assumptions of normal distribution (Shapiro–Wilk test) and homogeneity of variances (Levene’s test), the nonparametric Kruskal–Wallis test, followed by multiple comparisons of mean ranks using Dunn’s post hoc test, was used to identify significant differences at *p* ≤ 0.05 (Statistica 7.1 CZ).

The heat map was constructed using a freely available web server, “Heatmapper”, which was developed by Babicki et al. [20]. Before creating the heatmap, the concentration data for each phenolic compound was normalized using Z-score transformation.

## 3. Result and Discussion

### 3.1. Polyphenol Composition in Starting Materials

**Figure 1:**
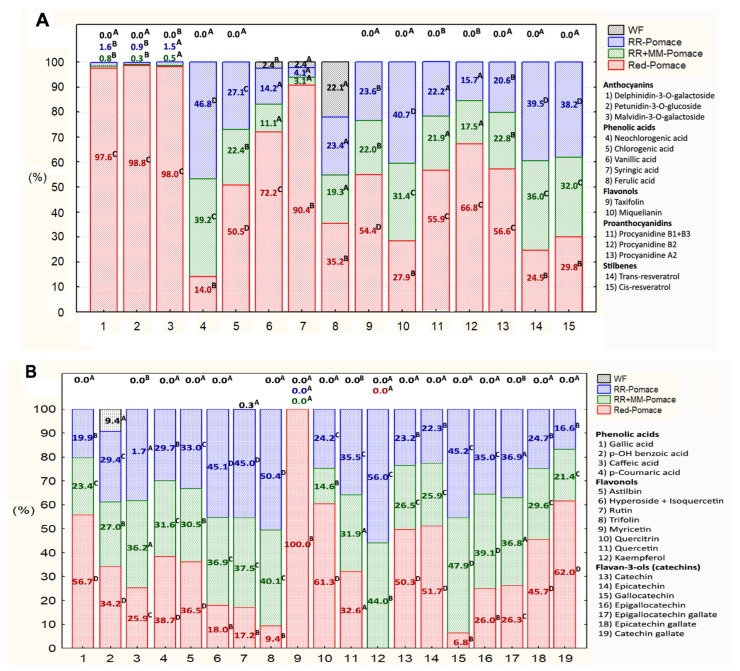
Relative comparison of 34 identified fPHEs in three types of grape pomace and white flour (WF) detected by LC-MS/MS in positive (**A**): ESI+ and negative (**B**): ESI− ionization modes. Values with a different letters are statisticaly significant at *p* ≤ 0.05 (Tukey HSD test). White pomaces: Rhine Riesling (RR); Rhine Riesling (RR) and Moravian Muscat (RR + MM). Red pomace: Saint Laurent and André varieties.

The relative contents of 34 detected free phenolic compounds (fPHEs) in the initial samples of three grape pomace types and white wheat flour are shown in Figure 1, whereas their absolute contents are summarized in the Supplementary Table S2. Two pairs of compounds—procyanidin B1 + B3 and hyperoside + isoquercetin were co-detected as single peaks by LC-MS, due to overlapping retention times of the isomeric compounds. The analysis revealed significant differences among the materials: while white flour contained only six fPHEs, grape pomace exhibited a broader spectrum of fPHEs with orders of magnitude higher concentrations, except for ferulic acid (3.2 µg/g), whose concentration was comparable to that found in flour.

Abouelenein et al. [6] detected 32 phenolic compounds (PHEs) primarily in pomace from red grape varieties. Our quantification represents an analytical selection compared to Rocchetti et al. [21], who identified up to 190 polyphenols in raw pomace using untargeted UHPLC-QTOF analysis.

In agreement with Abouelenein et al. [6], the highest content of fPHEs in our study was observed in pomace from a red grape blend, dominated by malvidin-3-O-galactoside (3713 µg/g) and petunidin-3-O-glucoside (26,242 µg/g), with concentrations orders of magnitude higher than other PHEs. The trace presence of myricetin (0.7 µg/g) was unique to red pomace. Pomace from white grape varieties showed higher concentrations of catechin and flavonols, particularly hyperoside + isoquercetin (390–476 µg/g) and miquelianin (527–684 µg/g), with kaempferol (2.7–3.5 µg/g) being a specific PHE for white varieties.

These differences highlight the significant varietal variability in secondary metabolite composition (Figure 1 and Supplementary Table S2). At the same time, processing conditions are likely to play a key role. For instance, Peixoto et al. [22], using LC-MS, did not detect any catechin in red grape pomace extract and found only trace amounts of epicatechin. The sum of seven detected anthocyanins in the extract was very low (7.9 µg/mL). In comparison, the total phenolic acids (TPA) in the same samples reached 1924 µg/mL. Khalangre et al. [23] reported malvidin-3-O-glucoside content in red pomace up to 15,503 µg/g (dry weight), while Abouelenein et al. [6] reported 4570 µg/g. In white grape pomace, Abouelenein et al. [6] identified delphinidin-3,5-diglucoside as the only anthocyanin (65.9 µg/g). These differences are likely attributable to the distinct technological processes used for red, white, and rosé wine production. Prolonged maceration of pressed grapes in red wine production allows for significantly higher release of polyphenols, including anthocyanins, into the must. Therefore, processing conditions should always be reported when describing the material, enabling more objective comparisons of PHE content.

### 3.2. Changes in Phenolic Compound Content During Bakery Processing with Grape Pomace Addition

**Figure 2:**
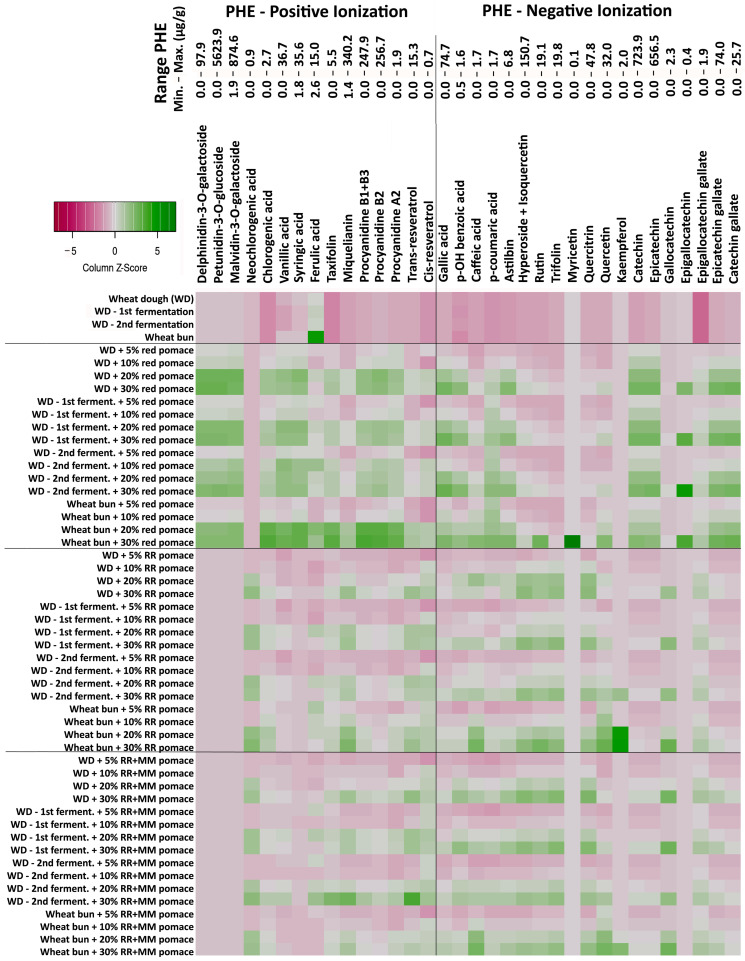
Heatmap showing changes in the content of the complete spectrum of 34 detected free phenolic compounds (fPHEs) in wheat flour and its mixtures with three types of grape pomace (5, 10, 20, and 30%) during the different stages of the baking process. White pomaces: Rhine Riesling (RR); Rhine Riesling (RR) and Moravian Muscat (RR + MM). Red pomace: Saint Laurent and André varieties.

The heatmap (Figure 2) illustrates the relative (standardized) changes in the content of 34 detected fPHEs during the bakery processing with varying levels of grape pomace from red and white varieties. Absolute mean values for the examined combinations are provided in the Supplementary (Supplementary Tables S3–S8).

In the control wheat dough without pomace, mainly free phenolic acids (fPHAs) typical for the wheat matrix were identified (e.g., ferulic acid 5.3 µg/g; vanillic acid 3.8 µg/g; syringic acid 2.6 µg/g; ESI+) as well as trace amounts of rutin (0.4 µg/g; ESI−) and p-hydroxybenzoic acid (0.5 µg/g). Compared to the raw flour, wheat dough and the final bun also showed very low levels of malvidin-3-O-galactoside (1.9–7.9 µg/g).

After baking the control bun, ferulic acid increased more markedly to 15.0 µg/g, and the highest concentration of malvidin-3-O-galactoside was observed (7.9 µg/g). The observed increase in ferulic acid in the final bun can be primarily attributed to the release of its bound forms induced by enzymatic and thermal hydrolysis. Similar increases in ferulic and vanillic acids have been reported by Skrajda-Brdak et al. [9].

From a general perspective, even a 5% addition of grape pomace resulted in an increase of approximately 26 out of 34 fPHEs in most intermediates and the final bun. With higher proportions of pomace in the wheat mixture, the content of the monitored fPHEs in both intermediates and the final bun increased further (Figure 2, Supplementary Tables S3–S8).

The addition of red pomace led mainly to a significant increase in anthocyanins, already evident at 5% addition compared to the control dough (e.g., petunidin-3-O-glucoside). A significant increase at the 5% level of red pomace was also observed for procyanidin B2, catechin, and epicatechin (Supplementary Tables S3 and S6). Unlike the anthocyanin petunidin-3-O-glucoside, whose content in the final bun was lower than in the raw dough, these fPHEs showed similar or, in the case of procyanidins, significantly higher concentrations in the final product compared to the processed dough with the same pomace level.

The addition of white grape pomace (RR, RR + MM) primarily increased the content of quantitatively significant fPHEs, such as (epi)catechins, procyanidins, and flavonols, including miquelianin. Statistically significant differences compared to the wheat dough were most frequently observed at 20–30% pomace addition (Supplementary Tables S7 and S8). These fPHEs generally remained stable or showed only slight decreases throughout the processing stages and in the final bun.

An exception was miquelianin, which reached its highest concentration in the final bun at all levels of white pomace addition from the RR variety. In contrast, the higher procyanidin levels observed in the final bun compared to the dough with red pomace were not confirmed with white pomace additions.

A significant increase in individual PHEs (catechin, epicatechin, quercetin, kaempferol, etc.) with increasing pomace content in flour has also been reported for baked cakes by Nakov et al. [1]. A similar effect of increasing total polyphenol content and antioxidant activity with progressive pomace addition in flour for a fermented and baked snack was confirmed by Alshawi [2].

#### Dominant Free Phenolic Compounds of Grape Pomace and Their Changes During Bakery Processing

**Figure 3:**
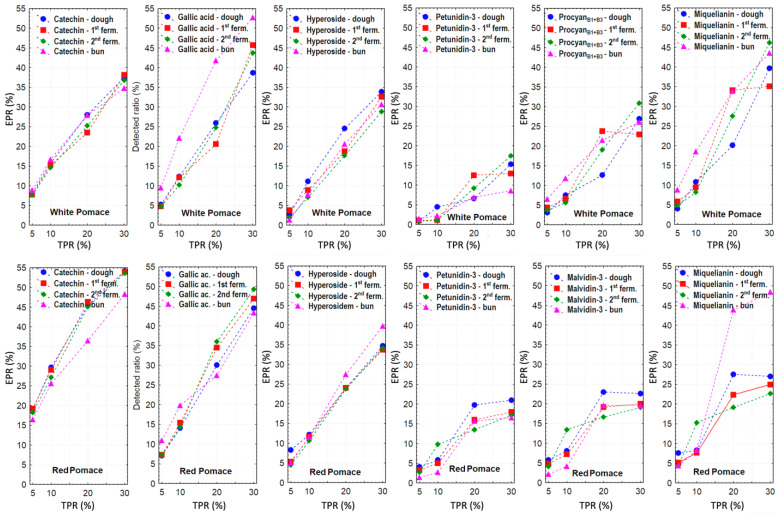
Comparison of theoretical (TPR) and equivalent pomace ratios (EPR) calculated for selected major PHEs across the dough, fermentation, and baking stages. White pomaces: Rhine Riesling (RR); Rhine Riesling (RR) and Moravian Muscat (RR + MM). Red pomace: Saint Laurent and André varieties. Mean value for white pomaces were calculated from the results obtained for RR and RR + MM samples. Hyperoside represents hyperoside + isoquercetin.

The evaluation focused on fPHEs exhibiting the highest concentrations in the original grape pomaces and representing chemically distinct phenolic groups, with their relative changes during fermentation and baking assessed through the equivalent pomace ratio (EPR) in relation to the theoretical pomace ratio (TPR) as described in the Materials and Methods. Trends in their relative concentrations are presented in Figure 3 and Supplementary Tables S9–S12.

In total, seven (fPHE) were included in this analysis, five of which were common to both white and red pomaces—catechin, gallic acid, hyperoside + isoquercetin, petunidin-3-O-glucoside, and miquelianin. Another major anthocyanin, malvidin-3-O-galactoside, was evaluated exclusively in red pomace samples. For white pomaces, the assessment was complemented by procyanidin B1 + B3. Given the similarity in their composition, values for the white pomaces (RR and RR + MM) were averaged.

As shown in Figure 3, EPR levels of selected fPHEs fluctuated around their theoretical values during processing. The closest agreement between EPR and TPR was observed for hyperoside + isoquercetin in both types of pomace. Higher EPR levels were observed for catechin, particularly gallic acid and miquelianin, suggesting their release from bound forms during fermentation and baking, in agreement with literature on phenolic acids (PHAs) in cereal matrices [24,25].

Nevertheless, published results are not entirely consistent. For example, in bread containing 25% quinoa, only two PHAs (hydroxybenzoic and rosmarinic acids) increased (64% and 435%, respectively), while the others decreased. Conversely, seven of the 13 detected flavonoids increased, with very high relative increases observed for apigenin (876%), kaempferol (1304%), luteolin (580%), and quercetin (4762%) in wheat–quinoa breads [26]. Nakov et al. [1] reported that in pomace-enriched cakes, flavonoid and PHAs levels in the final product were considerably lower than expected from their original content in the pomace.

In our study, the most pronounced decreases (EPR < TPR) were observed for anthocyanins (malvidin-3-O-galactoside and petunidin-3-O-glucoside). In red pomace, which contained these anthocyanins at much higher concentrations, EPR was approximately one-third lower than TPR, whereas in white pomaces the reduction exceeded 50% (Figure 3). Lower levels were already apparent in the processed dough and during its fermentation, indicating that the decrease was not solely due to thermal degradation during baking, as also confirmed by Khalangre et al. [23]. Despite this, anthocyanins remain the most abundant PHE group in the final baked product due to their high concentrations in the raw materials.

Khalangre et al. [23] further highlight that thermal degradation affects not only anthocyanins but also total flavonoids (TAF). The observed increases in some PHE can mainly be attributed to the release from bound forms. According to Rocchetti et al. [21], only about 9% of PHEs in pomace are bound. This likely indicates that the proportion of bound anthocyanins is relatively lower than that of free forms. Therefore, their potential release during processing is insufficient to compensate for the degradation of free anthocyanins, which, alongside their intrinsic lability, may further explain their decline during processing.

Supplementary Tables S9–S12 provide a comparison of EPR and TPR values for all remaining fPHEs throughout the baking process. PHAs and selected catechin derivatives showed higher-than-expected levels, whereas more stable fPHEs included astilbin, rutin, trifolin, and procyanidin B2. Conversely, some fPHEs decreased during processing, such as delphinidin-3-O-galactoside and gallocatechin.

Extremely high EPR values (9200–10,600%) were observed for quercitrin in combination with 30 % white pomace across all four processing stages (Supplementary Table S10), reflecting its initially very low content. With 30% red pomace addition, the EPR increase for quercetin was approximately ten times lower (Supplementary Table S9) compared to the average of the white pomaces. Free ferulic acid also exhibited a substantial (several-fold) increase during fermentation and after baking. However, this increase, including that of other PHAs, is not consistent with the findings reported by Gil et al. [26]. In their study, four PHAs (vanillic, p-coumaric, ferulic, and 2,4-dihydroxybenzoic acids) showed reductions of 53–65 % after baking wheat–quinoa blends.

These findings highlight that a detailed description of the applied processing conditions, including the characterization of bound PHE forms in the individual components of bakery mixes, is essential for understanding PHE content changes during the baking process. 

### 3.3. Rheological Behavior of Wheat Flour Supplemented with Grape Pomace

**Figure 4:**
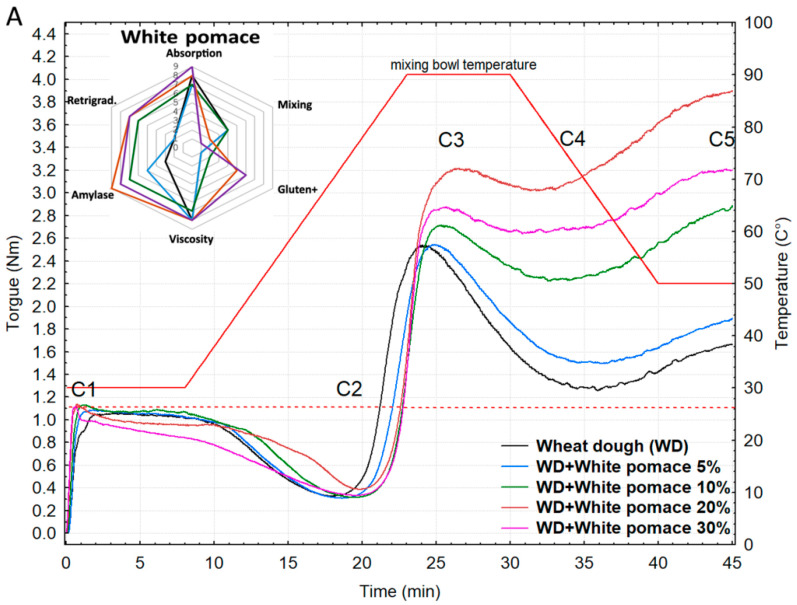

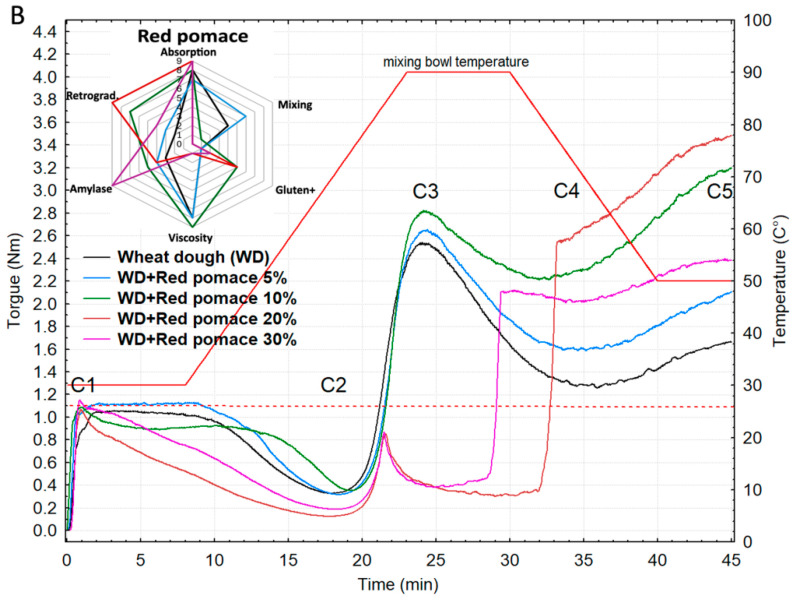
(**A**,**B**) Rheological profiles of wheat flour and composite mixtures with three types of grape pomace added at 5, 10, 20, and 30%. (**A**)—White pomaces: Rhine Riesling (RR) and Rhine Riesling + Moravian Muscat (RR + MM). (**B**)—Red grape pomace (Saint Laurent and André).

Rheological recordings obtained using a Mixolab instrument showed progressive changes in dough rheology with the replacement of wheat flour by pomace at levels of 5–30%. This effect was observed for both white grape pomaces (Riesling: RR and Riesling + Moravian Muscat: RR + MM) and the red grape pomace blend. Due to the very similar rheological profiles of the two white pomaces, the graph presents their averaged data (Figure 4A,B). Detailed significant rheological parameters for all three grape pomaces are provided in the Supplementary Table S13.

The addition of both red and white pomaces affected individual rheological parameters in a specific manner. For instance, water absorption and dough stability were only slightly influenced by white pomace additions up to 10%, and the corresponding rheological values remained largely stable. Similarly, the C2 parameter showed minimal variability for white pomace additions from 5% to 30%, with only a slight increase at 20% and 30%. A comparable rise in C2 (gluten weakening) with 10–20% pomace additions was also reported by Lou et al. [27], who explained this phenomenon by interactions of polyphenols with gluten, highlighting a trade-off between their weakening effect due to disruption of disulfide bonds and reinforcement of gluten strength via other interactions.

However, a dramatic decrease in the C2 parameter to 0.13 Nm and 0.19 Nm was observed with 20% and 30% red grape pomace, respectively. The higher PHE content in red grape pomace compared to white likely leads to substantial disruption of disulfide bridges in gluten at these inclusion levels, resulting in dough softening. Overall, the red pomace blend had a stronger effect on the protein-related part of the rheological curve, already evident at the 10% inclusion level (Figure 4B).

For both types of pomace, dough development time (C1) decreased markedly. In the control flour, development time was 3.97 min, while a 5% addition of white pomaces (RR and RR + MM) reduced it to 1.43 min and 1.82 min, respectively, and red pomace to 2.18 min, with further reductions at higher inclusion levels (Figure 4A,B, Supplementary Table S13). This effect is likely due to the higher fiber and simple sugar content in the pomace, enhancing hydration and accelerating dough development. A gradual increase in water absorption with Corvina pomace was also reported by Tolve et al. [28], who, however, did not observe reduced dough development time. These authors noted a trend toward increased dough stability with higher pomace content. Tao et al. [11] similarly reported increases in farinograph stability, the Mixing Tolerance Index (MTI), and farinograph quality number up to 1% isolated anthocyanins. In our study, this effect was observed only at 5% pomace, while 10% red and 20% white pomaces decreased dough stability (Figure 4A,B, Supplementary Table S13).

Starch-related parameters (C3—maximum starch gelatinization, C4—starchy gel stability, C5—retrogradation) mainly increased with white pomaces. Maximum starch gel viscosity (C3) in control flour was 2.54 Nm, rising to 3.28 Nm at 20% and 2.94 Nm at 30% Riesling pomace. Similar increases were observed for C4 and C5, indicating faster starch recrystallization and a tendency for quicker bread staling (Figure 4A). As far as we are aware, no comparable comprehensive evaluations of starch-related rheology using Mixolab have been reported, highlighting the novelty of the present study.

Beyond phenolic–protein/starch interactions, starch gelatinisation and gel stability are also influenced by non-phenolic components of grape pomace. The high content of insoluble fiber and pectins competes with starch for available water, alters hydration kinetics, and may limit granule swelling and gelatinisation [29]. Together with the physical dilution of the starch fraction and the presence of seed lipids and simple sugars, these water-binding and matrix-modifying effects plausibly contribute to the modulation of starch-phase parameters [28]. While white pomaces generally increased C3 and C4 values, the sharp reductions observed for red pomace at higher substitution levels (≥20%) may reflect the combined impact of intense water competition and matrix disruption alongside phenolic interactions. Importantly, these mechanisms operate together, and their relative weight likely rises with pomace level.

For red grape pomace, the progression of C3 and C4 parameters up to a 10% addition was similar to that observed for both white pomaces. However, at 20% addition, C3 and C4 values sharply decreased to 0.85 and 0.30 Nm, respectively. A pronounced increase in rheological resistance was observed only at the end of the analysis, primarily during the cooling of the kneaded dough, when the C5 parameter again exceeded the C5 value of the control sample (Figure 4B). This behavior is likely due to the very high concentration of phenolic compounds, particularly anthocyanins, interacting with starch chains and competing for water, which disrupted starch swelling and gelatinization. Consequently, the peak viscosity of the starch gel (C3) and its stability (C4) decreased. During cooling, similarly to the white pomace samples, integrated polyphenols bound more water than starch alone, accelerating starch molecule aggregation and leading to higher retrogradation.

The potential direct relationship between the rheological parameters measured by Mixolab and changes in the content of selected significant fPHEs in processed wheat dough with defined proportions of grape pomace was investigated by the correlation analysis (Table 2). Two correlation coefficients were calculated for each rheological parameter: one including all three grape pomace types and another based only on the two white pomaces. This approach was chosen because the red pomace was represented by a single sample, providing fewer data points for reliable correlation.

The results confirmed several strong positive and negative correlations (r ≥ 0.75/–0.75) between the monitored fPHEs and parameters of both the protein and starch phases of the dough (Table 2). For instance, correlations between miquelianin, procyanidins B1 + B3, and gallic acid and the absorption and C1 parameters can primarily be attributed to the higher fiber content, which increases water-binding capacity in the mixture and shortens dough development. Conversely, the strong correlations observed between starch phase parameters (C3, C4, and C5) and the monitored fPHEs, particularly from white grape pomaces, suggest possible interactions between phenolic compounds and wheat starch. An increasing proportion of the studied phenolic compounds thus enhanced starch viscosity (C3) and improved starch gel stability (C3–C4). Similar findings were reported by Zhu et al. [30] in a study investigating the effects of 25 PHEs (phenolic acids, flavonoids, coumarins, stilbenes, and tannins) on isolated starch using RVA analysis. Specific effects were observed on peak viscosity, while all tested phenolic compounds significantly increased gel stability during heating (breakdown), with chlorogenic acid having the strongest effect [30].

This corresponds to the observed increases in C3 and C4 parameters with white pomace addition (5–30%) and up to 10% for red pomace (Supplementary Table S13). According to Zhu et al. [30], these effects are mediated by the functional group composition of the phenolics, which interact with amylose and amylopectin through hydrogen bonding and van der Waals forces. The increasing C5 (retrogradation) reflects the ability of phenolic compounds, together with fiber, to bind more water than starch alone, accelerating starch molecule aggregation and enhancing retrogradation.

Published analyses of RVA starch with added complex grape pomace remain somewhat controversial. Lou et al. [27] and Alshawi [2] indicated that the addition of grape pomace generally reduced peak viscosity, final viscosity, and setback viscosity, likely due to the lower starch content and absence of gluten in the pomace, resulting in weaker gel formation and lower retrogradation. However, Nakov et al. [1] reported opposite RVA trends: additions of 0–8% pomace increased peak viscosity and starch retrogradation (setback), while breakdown decreased significantly from 6% pomace, which aligns better with our C3 and retrogradation findings in dough. According to Nakov et al. [1], the increased viscosity may be attributed to the high soluble fiber content in grape pomace, especially pectin, along with lipids in grape seeds, which can interact with other hydrophobic components (e.g., gluten), enhancing starch viscosity and slowing gelatinization. The potential influence of PHEs and their interactions with wheat starch was not directly addressed in this study.

Our correlation findings thus support the view that rheological changes are linked to the distinct phenolic profiles of white and red grape pomaces. White pomaces, rich in flavonols and phenolic acids, showed positive associations with starch-phase parameters (C3–C5), while red pomace, dominated by anthocyanins, exhibited inconsistent or negative trends at higher substitution levels. This suggests that flavonols and phenolic acids may support starch gel stability, whereas high anthocyanin loads could compete for water and disrupt gelatinisation. However, these interpretations remain indicative.

Therefore, a follow-up study using corresponding compositions and concentrations of the previously selected isolated PHEs detected in grape pomace would be appropriate to unequivocally confirm their effect on the rheological properties of proteins and starch in processed dough, without the influence of other interacting components.

#### Shape and Physical Profiles of Wheat Buns Containing Grape Pomace

The impact of grape pomace substitution on the physical and dimensional properties of wheat buns is summarized in Table 3, with visual documentation provided in Supplements (Supplementary Figure S1).

Even a 5% addition of either white or red grape pomace significantly altered bun shape and physical characteristics. Increasing pomace levels progressively reduced bun volume, height, shape ratio, bread yield, and specific volume, while bun width remained relatively stable, showing only a slight decline at 20% pomace. At 5% addition of grape pomace, the measured parameters decreased by 3–19% compared to the standard wheat bun. A 10% addition of grape pomace caused a marked reduction to approximately 53–54% of the control values, and further increases (30%) lowered them to roughly one-third of the wheat control.

Statistical analysis indicated consistent effects of white and red pomace, with no significant differences between both types (Table 3). Notably, rheological drops in the starch phase at 20–30% red pomace (Figure 4B) did not significantly influence bun shape or physical properties, although they may affect dough processing (kneading) and sensory attributes such as crumb texture and shelf-life.

These changes are primarily attributed to gluten weakening due to flour substitution, as confirmed by shorter dough development time (C1) and reduced stability (Figure 4A,B). A similar effect on bread volume and specific volume was reported by Tolve et al. [28], who also observed that pomace additions up to 10% did not significantly affect the overall acceptability of enriched products. The observed reduction in bun volume and alteration in shape following the substitution of wheat flour with grape pomace cannot be attributed solely to the weakening of the gluten matrix.

In addition to gluten weakening, several concurrent factors are likely to contribute to the reduction in bun volume and changes in shape. One factor is the limited availability of water for wheat proteins due to the high water-binding capacity of dietary fiber. Verbeke et al. [31] demonstrated that fiber addition significantly increases water absorption and affects dough development and stability, particularly at higher fiber concentrations. Another factor is the physical disruption of gas cell formation and stability by insoluble fiber particles. Ma et al. [32] reported that fiber particles can destabilize gas cell interfaces, limit dough expansion, and reduce the specific volume of baked goods.

Interactions between PHEs and gluten proteins may also play an important role. Schefer et al. [33] described that PHAs, particularly ferulic acid, can affect gluten protein conformation, leading to partial denaturation or cross-linking, thereby disrupting the dough network structure. Furthermore, Šporin et al. [34] suggested that PHEs from grape pomace may inhibit yeast growth, which could result in lower gas production and a denser crumb structure. Thus, a comprehensive understanding of these mechanisms will be essential for optimizing recipes and technological processes when using wine by-products in baking.

### 3.4. Interactions of Phenolic Compounds from Grape Pomace with Proteins and Starch in Wheat Buns

To investigate the interactions of phenolic compounds (PHEs) from grape pomace with starch and proteins, wheat flour mixtures containing 30% white or red grape pomace were analyzed using FTIR spectroscopy. Differential spectra were obtained by subtracting the spectrum of the wheat bun from those of the pomace-enriched buns, highlighting the specific effects of grape pomace.

**Figure 5:**
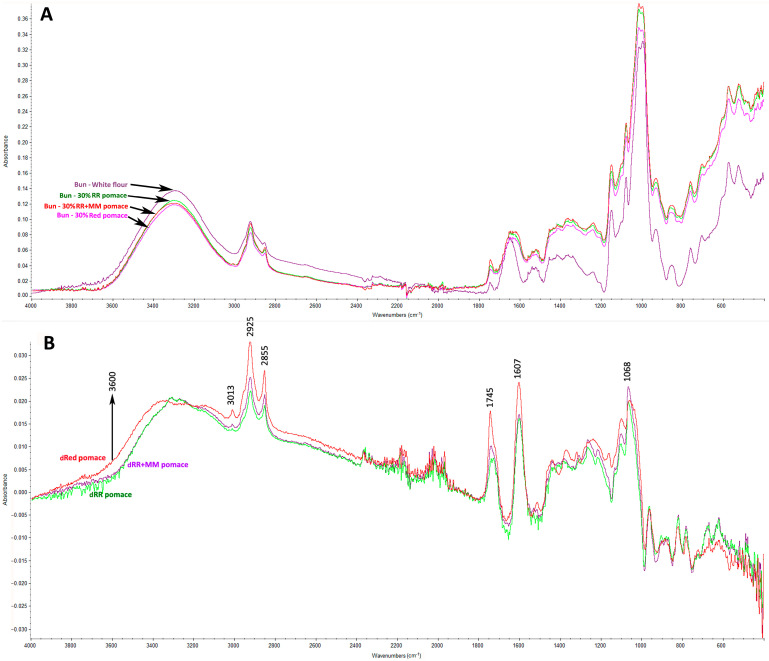
(**A**) FTIR spectra of wheat bun and wheat buns enriched with 30% grape pomace, (**B**) differential spectra obtained by subtracting the spectrum of the wheat bun from those of the pomace-enriched buns. White pomaces: Rhine Riesling (RR); Rhine Riesling (RR) and Moravian Muscat (RR + MM). Red pomace: Saint Laurent and André varieties.

Figure 5A,B illustrates these results: (A) FTIR spectra of the wheat bun and buns enriched with 30% grape pomace, and (B) differential spectra obtained by subtraction. The differential spectra revealed significant changes in several key regions compared to the plain wheat bun. Two prominent peaks at 1607 cm^−1^ and 1745 cm^−1^, particularly pronounced in the mixture with red grape pomace, corresponded to bending vibrations of aromatic C = H rings, indicative of PHEs such as anthocyanins [35,36]. These compounds also affected the Amide I (1600–1700 cm^−1^) and Amide II (1500–1600 cm^−1^) regions, suggesting interactions with the protein fraction of wheat flour [37]. Phenolics can form hydrogen bonds and covalent interactions with gluten peptide chains, altering gluten secondary structure and partially disrupting the gluten network [38]. In our study, this was reflected in rheological measurements as a shorter dough development time (C1) and reduced dough stability (see Section 3.3).

A second broad region of spectral differences was observed between 1400 and 900 cm^−1^, with a maximum at 1068 cm^−1^. This region extends into the Amide III band (1200–1320 cm^−1^), indicating potential further interactions with wheat proteins [38]. The 1200–900 cm^−1^ range also corresponds to changes in C–O–C and C–O absorption bands of polysaccharides [39,40]. These spectral shifts can be attributed to non-covalent interactions, mainly hydrogen bonding, between hydroxyl groups of phenolics and glycosidic linkages in starch, resulting in structural reorganization of the starch matrix. Additional spectral differences appeared as two peaks at 2855 cm^−1^ and 2925 cm^−1^, corresponding to C–H stretching vibrations of glucose units in starch. In native plant starches, however, only a single peak around 2930 cm^−1^ is typically observed [39,40]. Finally, a broad region between 3000 and 3600 cm^−1^, primarily associated with O–H stretching vibrations, was identified. In both previously mentioned regions, red grape pomace exhibited broader and more intense signals, suggesting stronger hydrogen bonding and tighter interactions between phenolics and the polysaccharide network. These differences may explain the observed rheological changes (see Section 3.3).

Beyond protein interactions, the spectral differences in the polysaccharide region (1200–900 cm^−1^) and the intensified O–H stretching band (3000–3600 cm^−1^) indicate strong hydrogen bonding between phenolics and starch. These interactions may affect water availability and, consequently, influence gelatinisation and retrogradation processes. These trends correlate with Mixolab results: white pomace, rich in flavonols and phenolic acids, tended to show higher C3 and C4 values (indicative of enhanced starch gel stability), whereas red pomace at ≥20% was associated with reduced C3/C4 and higher C5 (accelerated retrogradation), likely due to water competition and partial matrix disruption. While other components of grape pomace (e.g., fiber, lipids) may also contribute to these effects, the observed patterns support the hypothesis that phenolic compounds play a significant role in modifying both protein and polysaccharide fractions of the dough.

## 4. Conclusions

Despite some losses, particularly of anthocyanins, the concentrations of most tested fPHEs throughout processing and in the final baked products corresponded to their theoretically expected levels and in some cases even exceeded them. For several compounds, including catechin, epicatechin, and gallic acid, their levels were notably higher than expected. These results confirm that incorporating grape pomace is an effective strategy to enhance the content of free bioactive compounds in baked products, even at relatively low replacement levels (5%).

From a technological perspective, the addition of grape pomace also significantly affects dough rheology as well as the final shape and physical structure of the buns. At low substitution levels (5%), only a slight reduction in dough development time and minor changes in water absorption were observed, which slightly impaired the buns’ physical and shape properties. Higher inclusion levels (20–30%), particularly of red grape pomace rich in anthocyanins, result in substantial disruption of the gluten network and a pronounced reduction in starch gelatinization. Nevertheless, these higher levels of grape pomace could potentially be considered for non-leavened baked goods, such as cookies or crackers, where firmer texture and faster retrogradation are less critical.

## Figures and Tables

**Table 1:** Chromatographic and mass spectrometric parameters for the selected PHEs (in alphabetical order, and including verapamil and probenecid as internal standards) analyzed in grape pomace extract by UHPLC-HRMS/MS analysis (positive or negative mode). Quantification and confirmation ions were obtained at NCE.

Compound	Elemental Composition	Polarity	NCE *	Rt (min)	Precursor ion (*m*/*z*)	Quantification ion (*m*/*z*)	Confirmation ion (*m*/*z*)
Astilbin	C_21_H_22_O_11_	negative	20	8.90	449.1089	151.0026	285.0404
Caffeic acid	C_9_H_8_O_4_	negative	40	6.40	179.0350	135.0439	117.0334
Catechin	C_15_H_14_O_6_	negative	30	5.38	289.0718	245.0814	109.0281
Catechin gallate	C_22_H_18_O_10_	negative	20	7.93	441.0828	169.0132	289.0708
Chlorogenic acid	C_16_H_18_O_9_	positive	20	5.94	355.1024	163.0389	145.0281
Cis-resveratrol	C_8_H_8_O_3_	positive	32	10.45	229.0859	135.0441	119.0499
Delphinidine-3-O-galactoside	C_21_H_20_O_12_	positive	30	6.12	465.1028	303.0487	257.0443
Epicatechin	C_15_H_14_O_6_	negative	30	6.66	289.0718	245.0814	109.0281
Epicatechin gallate	C_22_H_18_O_10_	negative	20	7.54	441.0828	169.0132	289.0708
Epigallocatechin	C_15_H_14_O_7_	negative	35	5.18	305.0667	125.0232	219.0656
Epigallocatechin gallate	C_22_H_18_O_11_	negative	20	6.24	457.0771	169.0132	305.0663
Ferulic acid	C_10_H_10_O_4_	positive	45	8.23	195.0643	177.0545	145.0283
Gallic acid	C_7_H_6_O_5_	negative	45	2.76	169.0123	125.0232	97.0282
Gallocatechin	C_15_H_14_O_7_	negative	35	3.80	305.0667	125.0232	167.0340
Gallocatechin gallate	C_22_H_18_O_11_	negative	20	6.89	457.0771	169.0132	305.0663
*p*-Hydroxybenzoic acid	C_7_H_6_O_3_	negative	60	5.46	137.0244	93.0332	65.0383
Hyperoside	C_21_H_20_O_12_	negative	30	9.23	463.0882	300.0267	271.0244
Isoquercetin	C_21_H_20_O_12_	negative	30	9.31	463.0882	300.0267	271.0244
Kaempferol	C_15_H_10_O_6_	negative	70	12.20	285.0405	185.0598	93.0332
Malvidine-3-O-galactoside	C_23_H_24_O_12_	positive	20	7.54	493.1341	331.0799	315.0496
Miquelianin	C_21_H_18_O_13_	positive	15	9.19	479.0820	303.0498	229.0495
Myricetin	C_15_H_10_O_8_	negative	40	9.95	317.0303	151.0025	178.9977
Neochlorogenic acid	C_16_H_18_O_9_	positive	20	4.63	355.1024	163.0389	145.0281
Petunidine-3-O-glucoside	C_22_H_22_O_12_	positive	20	6.92	479.1184	317.0659	302.0425
Probenecid (intern. standard)	C_13_H_19_NO_4_S	negative	30	12.39	284.0957	198.0584	240.1057
Procyanidine A2	C_30_H_24_O_12_	positive	10	7.73	577.1341	425.0858	287.0540
Procyanidine B1	C_30_H_26_O_12_	positive	20	4.73	579.1497	127.0389	409.0907
Procyanidine B2	C_30_H_26_O_12_	positive	20	5.71	579.1497	127.0389	409.0907
Procyanidine B3	C_30_H_26_O_12_	positive	20	4.65	579.1497	127.0389	409.0907
Quercetin	C_15_H_10_O_7_	negative	40	11.21	301.0354	151.0024	178.9975
Quercitrin	C_21_H_20_O_11_	negative	30	10.12	447.0933	300.0270	271.0239
Rutin	C_27_H_30_O_16_	negative	40	9.28	609.1461	300.0267	271.0241
Syringic acid	C_9_H_60_O_5_	positive	30	6.89	199.0596	140.0465	155.0700
Taxifolin	C_15_H_12_O_7_	positive	20	8.01	305.0656	259.0596	153.0180
*trans*-*p*-Coumaric acid	C_9_H_8_O_3_	negative	10	7.73	163.0388	119.0492	91.0547
Trans-resveratrol	C_8_H_8_O_3_	positive	32	9.45	229.0859	135.0441	119.0499
Trifolin	C_21_H_20_O_10_	negative	32	9.88	447.0937	284.0326	255.0288
Vanillic acid	C_8_H_8_O_4_	positive	40	6.36	169.0496	111.0442	125.0597
Verapamil (intern. standard)	C_27_H_38_N_2_O_4_	positive	40	11.04	455.2902	165.0909	303.2066

NCE *: normalized collision energy.

**Table 2:** Spearman correlation analysis between quantitatively significant fPHEs in processed dough (WD) and Mixolab rheological parameters with increasing proportions of white and red grape pomaces in wheat flour (5, 10, 20, and 30%).

Parameters	Delphinidin–3–O–galactoside ^1^	Petunidin–3–O–glucoside	Malvidin–3–O–galaktoside	Miquelianin	Procyanidine B1 + B3	Gallic Acid	Hyperoside + Isoquercetin	Catechin
Absorption	0.36	0.61 * (0.80 **)	0.58 * (0.80 **)	0.77 ** (0.82 **)	0.75 ** (0.78 *)	0.81 ** (0.80)	0.69 ** (0.78 *)	0.64 * (0.75 *)
C1 (min)	−0.12	−0.51 (−0.84 **)	−0.25 (−0.31)	−0.84 ** (−0.91)	−0.75 ** (−0.99 **)	−0.81 ** (−0.98)	−0.88 ** (−0.95 **)	−0.66 * (−0.99 **)
Dough stability (min)	−0.24	−0.44 (−0.53)	−0.38 (−0.35)	−0.53 (−0.55)	−0.60 * (−0.63)	−0.64 * (−0.65)	−0.48 (−0.53)	−0.52 (−0.62)
C2 (Nm)	−0.41	−0.20 (0.30)	−0.14 (0.50)	0.11 (0.40)	−0.15 (0.50)	−0.10 (0.40)	0.24 (−0.40)	−0.12 (0.50)
C1–C2 (Nm)	0.41	0.20 (−0.30)	0.14 (−0.50)	−0.11 (−0.40)	0.15 (−0.50)	0.10 (−0.40)	−0.24 (−0.40)	0.12 (−0.50)
C3 (Nm)	−0.48	−0.05 (0.78 *)	−0.20 (0.45)	0.44 (0.83 **)	0.01 (0.78 *)	0.07 (0.77 *)	0.56 * (0.78 *)	−0.02 (0.80 **)
C4 (Nm)	−0.54	−0.10 (0.75 *)	−0.30 (0.32)	0.47 (0.83 **)	0.01 (0.83 **)	0.05 (0.82 **)	0.62 * (0.83 **)	−0.04 (0.85 **)
C3–C4 (Nm)	0.16	−0.28 (−0.75 *)	−0.02 (−0.29)	−0.85 ** (−0.83 **)	−0.49 (−0.85 **)	−0.54 (−0.83 **)	−0.92 ** (−0.95 **)	−0.39 (−0.87 **)
C5 (Nm)	0.06	0.45 (0.79 *)	0.25 (0.32)	0.77 ** (0.85 **)	0.53 (0.82 **)	0.63 * (0.83 **)	0.76 ** (0.85 **)	0.53 (0.85 **)

* Statistically significant at *p* ≤ 0.05; ** Statistically significant at *p* ≤ 0.01; Correlations shown in brackets apply only to the relationships between white grape pomace and Mixolab parameters. ^1^ Delphinidin-3-O-galactoside was not detected in white grape pomace.

**Table 3:** Shape and physical characteristics of wheat buns (WB) with different levels of white and red grape pomace substitution (Mean value ± SD).

Samples	Volume (mL)	Height (cm)	Width (cm)	Height/Width	Bread Yield (cm^3^/100 g WF ^1^)	Specific Volume (cm^3^/100 g bun)
Wheat bun (WB)	245 ± 1.7 ^d^	6.6 ± 0.2 ^d^	8.4 ± 0.2 ^c^	0.8 ± 0.0 ^b^	499.2 ± 3.4 ^e^	356.6 ± 2.4 ^d^
WB + 5% W. pomace	204 ± 21.1 ^c^	6.0 ± 0.2 ^cd^	8.2 ± 0.3 ^c^	0.7 ± 0.0 ^b^	413.9 ± 41.8 ^b^	291.8 ± 33.3 ^c^
WB + 10% W. pomace	133 ± 25.7 ^b^	5.1 ± 0.4	8.0 ± 0.3 ^bc^	0.6 ± 0.1 ^c^	265.9 ± 53.5 ^c^	189.1 ± 37.4 ^b^
WB + 20% W. pomace	95 ± 5.9 ^a^	3.6 ± 0.3 ^ab^	7.6 ± 0.1 ^ab^	0.5 ± 0.0 ^a^	194.4 ± 11.5 ^ab^	134.9 ± 7.6 ^a^
WB + 30% W. pomace	74 ± 5.4 ^a^	3.1 ± 0.3 ^a^	7.6 ± 0.4 ^ab^	0.4 ± 0.1 ^a^	155.8 ± 11.3 ^a^	107.1 ± 7.8 ^a^
WB + 5% R. pomace	213 ± 0.0 ^c^	5.7 ± 0.3 ^c^	8.5 ± 0.2 ^c^	0.7 ± 0.0 ^bc^	429.3 ± 15.2 ^de^	304.9 ± 6.9 ^cd^
WB + 10% R. pomace	130 ± 3.3 ^b^	4.1 ± 0.1 ^b^	8.0 ± 0.1 ^abc^	0.5 ± 0.0 ^a^	260.9 ± 6.7 ^bc^	189.6 ± 4.9 ^b^
WB + 20% R. pomace	90 ± 3.3 ^a^	3.4 ± 0.1 ^ab^	7.5 ± 0.2 ^ab^	0.5 ± 0.0 ^a^	185.9 ± 6.9 ^ab^	129.2 ± 4.8 ^a^
WB + 30% R. pomace	73 ± 0.0 ^a^	3.6 ± 0.1 ^ab^	7.3 ± 0.4 ^a^	0.5 ± 0.0 ^a^	159.1 ± 8.7 ^a^	111.2 ± 4.6 ^a^

Values with a different letters are statisticaly significant at *p* ≤ 0.05 (Tukey HSD test). ^1^ White flour or mixture with grape pomace. W. pomaces: White pomace—Rhine Riesling (RR); Rhine Riesling (RR) and Moravian Muscat (RR + MM). R. pomace: Red pomace—Saint Laurent and André varieties.

## Data Availability

The original contributions presented in the study are included in the article/Supplementary Materials, further inquiries can be directed to the corresponding authors.

## Supplementary Materials

The following supporting information can be downloaded at: https://www.mdpi.com/article/10.3390/foods14244256/s1, Figure S1. Wheat buns enriched with different proportions of white and red grape pomace (WF: Wheat flour; RED: Red grape pomace–Saint Laurent and André varieties; RR: White pomace–Rhine Riesling; RR + MM: White pomace–Rhine Riesling and Moravian Muscat). Table S1. Basic chemical composition of grape pomace samples. Table S2. Content comparison of 34 identified free phenolic compounds in three types of grape pomace and white flour (WF) detected by LC-MS/MS in positive (ESI+) and negative (ESI–) ionization modes. Table S3. Average content of phenolic compounds (µg/g) measured during processing of control wheat dough and dough with a defined proportion of red grape pomace (positive ionization, ESI+); Kruskal-Wallis test followed by Dunn’s post-hoc test. Table S4. Average content of phenolic compounds (µg/g) measured during processing of control wheat dough and dough with a defined proportion of white grape pomace–RR (positive ionization, ESI+); Kruskal-Wallis test followed by Dunn’s post-hoc test. Table S5. Average content of phenolic compounds (µg/g) measured during processing of control wheat dough and dough with a defined proportion of white grape pomace–RR + MM (positive ionization, ESI+); Kruskal-Wallis test followed by Dunn’s post-hoc test. Table S6. Average content of phenolic compounds (µg/g) measured during processing of control wheat dough and dough with a defined proportion of red grape pomace (negative ionization, ESI–); Kruskal-Wallis test followed by Dunn’s post-hoc test. Table S7. Average content of phenolic compounds (µg/g) measured during processing of control wheat dough and dough with a defined proportion of white grape pomace–RR (negative ionization, ESI–); Kruskal-Wallis test followed by Dunn’s post-hoc test. Table S8. Average content of phenolic compounds (µg/g) measured during processing of control wheat dough and dough with a defined proportion of white grape pomace–RR + MM (negative ionization, ESI–); Kruskal-Wallis test followed by Dunn’s post-hoc test. Table S9. Comparison of theoretical (TPR) and equivalent (EPR) pomace ratios for other phenolic compounds in red grape pomace during dough mixing, fermentation and baking (negative ionization, ESI–). Table S10. Comparison of theoretical (TPR) and equivalent (EPR) pomace ratios for other phenolic compounds in white grape pomaces (RR, RR + MM) during dough mixing, fermentation and baking (negative ionization, ESI–). Table S11. Comparison of theoretical (TPR) and equivalent (EPR) pomace ratios for other phenolic compounds in red grape pomace during dough mixing, fermentation and baking (positive ionization, ESI+). Table S12. Comparison of theoretical (TPR) and equivalent (EPR) pomace ratios for other phenolic compounds in white grape pomace during dough mixing, fermentation and baking (positive ionization, ESI+). Table S13. Rheological parameters of wheat dough supplemented with white grape pomace Riesling Rhine (RR), Riesling Rhine + Moravian Muscat (RR + MM), and red grape pomace Saint Laurent + André (RED)–Mixolab I.
