# Supplementary material for: Processing-Induced Changes in Phenolic Composition and Dough Properties of Grape Pomace-Enriched Wheat Buns
Source: Foods. 2025 Dec 10;14(24):4256. doi: 10.3390/foods14244256 (PMC12733168; doi:10.3390/foods14244256)
Supplement: Supplementary file 1 [file foods-14-04256-s001.zip › foods-3989938-supplementary.pdf]

Supplements

Table S1. Basic chemical composition of grape pomace samples

Pomace / Composition	Dry matter (%)	Crude Protein (%)	Glucose (%)	Fructose (%)	Fat (%)	Ash (%)	Crude Fiber (%)
Red pomace	92.6 ± 1.9	11.8 ± 0.2	2.7 ± 0.1	2.5 ± 0.1	5.0 ± 0.3	4.4 ± 0.2	11.8 ± 0.2
White pomace RR	88.1 ± 1.8	11.0 ± 0.2	5.0 ± 0.3	6.5 ± 0.3	5.4 ± 0.3	4.9 ± 0.3	9.1 ± 0.2
White pomace RR+MM	88.3 ± 1.8	10.9 ± 0.2	4.7 ± 0.2	6.3 ± 0.3	6.6 ± 0.3	4.6 ± 0.2	8.9 ± 0.2

Red pomace: Saint Laurent and André varieties, White pomaces: Rhine Riesling (RR); Rhine Riesling (RR) and Moravian Muscat (RR+MM)

Table S2. Content comparison of 34 identified free phenolic compounds in three types of grape pomace and white flour (WF) detected by LC-MS/MS in positive A: ESI$^+$ and negative B: ESI$^-$ ionization modes.

Phenolic composition (μg/g)	ESI	Wheat flour	White pomace RR	White pomace RR+MM	Red pomace
N		9	9	9	9
Delphinidin-3-O-galactoside	ESI$^+$	0.0 ± 0.0^a	7.5 ± 2.1^b	4.0 ± 0.2^b	462.3 ± 42.4^c
Petunidin-3-O-glucoside	ESI$^+$	0.0 ± 0.0^a	243.4 ± 113.0^b	72.5 ± 6.8^b	26242.7 ± 2012.8^c
Malvidin-3-O-galactoside	ESI$^+$	0.0 ± 0.0^b	56.2 ± 21.4^a	19.5 ± 1.7^a	3713.0 ± 342.1^c
Neochlorogenic acid	ESI$^+$	0.0 ± 0.0^a	1.7 ± 0.1^d	1.5 ± 0.1^c	0.5 ± 0.0^b
Chlorogenic acid	ESI$^+$	0.0 ± 0.0^a	2.8 ± 0.2^c	2.3 ± 0.1^b	5.1 ± 0.5^d
Vanillic acid	ESI$^+$	2.4 ± 1.9^b	14.0 ± 3.3^a	11.0 ± 1.7^a	71.3 ± 12.8^c
Syringic acid	ESI$^+$	2.1 ± 0.1^a	3.6 ± 0.4^a	2.8 ± 0.1^a	80.6 ± 7.0^b
Ferulic acid	ESI$^+$	3.2 ± 1.2^a	3.3 ± 0.2^a	2.8 ± 0.1^a	5.0 ± 0.5^b
Taxifolin	ESI$^+$	0.0 ± 0.0^a	5.2 ± 0.4^b	4.8 ± 0.4^b	11.9 ± 0.9^d
Miquelianin	ESI$^+$	0.5 ± 0.7^a	683.5 ± 39.0^d	526.5 ± 31.2^c	468.7 ± 43.8^b
Procyanidins B1+B3	ESI$^+$	0.0 ± 0.0^b	269.0 ± 14.4^a	266.2 ± 12.3^a	678.5 ± 66.6^c
Procyanidin B2	ESI$^+$	0.0 ± 0.0^b	171.4 ± 10.0^a	191.2 ± 10.0^a	728.2 ± 67.7^c
Procyanidin A2	ESI$^+$	0.0 ± 0.0^a	1.3 ± 0.1^b	1.5 ± 0.1^b	3.7 ± 0.3^c
Trans-resveratrol	ESI$^+$	0.0 ± 0.0^a	31.4 ± 2.2^d	28.6 ± 1.1^c	19.5 ± 1.9^b
Cis-resveratrol	ESI$^+$	0.0 ± 0.0^a	0.9 ± 0.0^d	0.8 ± 0.0^c	0.7 ± 0.0^b
Gallic acid	ESI$^-$	0.0 ± 0.0^a	55.3 ± 1.1^b	65.0 ± 1.1^c	157.4 ± 11.3^d
p-OH benzoic acid	ESI$^-$	0.4 ± 0.0^a	1.4 ± 0.1^c	1.3 ± 0.0^b	1.6 ± 0.1^d
Caffeic acid	ESI$^-$	0.0 ± 0.0^b	1.7 ± 0.0^a	1.6 ± 0.0^a	1.1 ± 0.1^c
p-Coumaric acid	ESI$^-$	0.0 ± 0.0^a	1.1 ± 0.0^b	1.1 ± 0.0^c	1.4 ± 0.1^d
Astilbin	ESI$^-$	0.0 ± 0.0^a	13.0 ± 0.2^c	12.0 ± 0.2^b	14.4 ± 0.6^d
Hyperoside + Isoquercetin	ESI$^-$	0.0 ± 0.0^a	476.3 ± 6.2^d	389.5 ± 9.1^c	190.5 ± 4.6^b
Rutin	ESI$^-$	0.2 ± 0.3^a	42.6 ± 0.7^d	35.5 ± 0.7^c	16.3 ± 0.7^b
Trifolin	ESI$^-$	0.0 ± 0.0^a	54.3 ± 0.8^d	43.2 ± 0.7^c	10.2 ± 0.5^b
Myricetin	ESI$^-$	0.0 ± 0.0^a	0.0 ± 0.0^a	0.0 ± 0.0^a	0.8 ± 0.2^b
Quercitrin	ESI$^-$	0.0 ± 0.0^a	0.6 ± 0.0^c	0.4 ± 0.0^b	1.5 ± 0.1^d
Quercetin	ESI$^-$	0.0 ± 0.0^b	33.3 ± 1.2^c	30.0 ± 1.8^a	30.6 ± 1.7^a
Kaempferol	ESI$^-$	0.0 ± 0.0^a	3.5 ± 0.3^a	2.7 ± 0.4^c	0.0 ± 0.0^b
Catechin	ESI$^-$	0.0 ± 0.0^a	633.3 ± 10.2^b	723.4 ± 12.9^c	1370.8 ± 90.1^d
Epicatechin	ESI$^-$	0.0 ± 0.0^a	534.4 ± 5.7^b	619.6 ± 12.4^c	1237.7 ± 79.0^d
Gallocatechin	ESI$^-$	0.0 ± 0.0^a	18.2 ± 0.5^c	19.3 ± 0.4^d	2.7 ± 0.4^b
Epigallocatechin	ESI$^-$	0.0 ± 0.0^a	2.7 ± 0.1^c	3.0 ± 0.2^d	2.0 ± 0.2^b
Epigallocatechin gallate	ESI$^-$	0.0 ± 0.0^b	3.5 ± 0.1^a	3.5 ± 0.1^a	2.5 ± 0.0^c
Epicatechin gallate	ESI$^-$	0.0 ± 0.0^a	44.9 ± 1.6^b	53.7 ± 1.2^c	83.1 ± 2.1^d
Catechin gallate	ESI$^-$	0.0 ± 0.0^a	14.1 ± 0.5^b	18.2 ± 0.5^c	52.7 ± 1.5^d

*Red pomace: Saint Laurent and André varieties, White pomaces: Rhine Riesling (RR); Rhine Riesling (RR) and Moravian Muscat (RR+MM)

**Values in individual rows with a different letters are statisticaly significant at $p \leq 0.05$ (Tukey HSD test).

Table S3. Average content of phenolic compounds (μg/g) measured during processing of control wheat dough and dough with a defined proportion of red grape pomace (positive ionization, ESI+); Kruskal-Wallis test followed by Dunn's post-hoc test

Processing / PHE (μg/g)	Delphinidin-3-O-galactoside	Petunidin-3-O-glucoside	Malvidin-3-O-galactoside	Neochlorogenic acid	Chlorogenic acid	Vanillic acid	Syringic acid	Ferulic acid	Taxifolin	Miquelianin	Procyanidine B1+B3	Procyanidine B2	Procyanidine A2	Trans-resveratrol	Cis-resveratrol
Max. standard. deviation[1]	10.7	738.2	126.0	0.0	0.3	5.0	6.1	1.4	1.1	33.2	33.0	37.3	0.2	1.3	0.2
Wheat dough (WD)	0.0^{a}	0.0^{a}	6.1^{a}	0.0^{a}	0.0^{a}	3.8^{a}	2.6^{a}	5.3^{a}	0.0^{a}	1.8^{a}	0.0^{a}	0.0^{a}	0.0^{a}	0.0^{a}	0.0^{a}
WD-1. fermentation	0.0^{a}	0.0^{a}	2.0^{a}	0.0^{a}	0.0^{a}	0.0^{a}	2.1^{a}	6.1^{a}	0.0^{a}	1.4^{a}	0.0^{a}	0.0^{a}	0.0^{a}	0.0^{a}	0.0^{a}
WD-2. fermentation	0.0^{a}	0.0^{a}	1.9^{a}	0.0^{a}	0.0^{a}	0.0^{a}	2.0^{a}	5.9^{a}	0.0^{a}	1.4^{a}	0.0^{a}	0.0^{a}	0.0^{a}	0.0^{a}	0.0^{a}
Wheat bun	0.0^{a}	0.0^{a}	7.9^{a}	0.0^{a}	0.6^{a}	6.2^{a}	2.7^{a}	15.0^{a}	0.0^{a}	1.5^{a}	0.0^{a}	0.0^{a}	0.0^{a}	0.0^{a}	0.0^{a}
WD+5% red pomace	19.9^{a}	**1099.6^{b}**	219.6^{a}	0.0^{a}	0.8^{a}	12.3^{a}	8.8^{a}	4.6^{a}	1.7^{a}	36.4^{a}	43.0^{a}	**50.7^{b}**	0.6^{a}	1.4^{a}	0.3^{a}
WD+10% red pomace	27.7^{a}	**1563.6^{b}**	307.8^{ab}	0.0^{a}	0.8^{a}	12.7^{a}	11.3^{a}	3.4^{a}	1.8^{a}	39.6^{a}	56.0^{b}	**67.0^{b}**	0.6^{a}	1.2^{a}	0.0^{a}
WD+20% red pomace	**91.8^{b}**	**5301.8^{b}**	**874.6^{b}**	0.0^{a}	**1.6^{b}**	**26.9^{b}**	27.0^{a}	6.0^{a}	**3.2^{b}**	131.9^{b}	**142.9^{b}**	**176.9^{b}**	**1.2^{b}**	**4.7^{b}**	0.5^{a}
WD+30% red pomace	**97.9^{b}**	**5623.9^{b}**	**859.6^{b}**	0.0^{a}	**1.6^{b}**	**24.9^{b}**	23.8^{a}	4.4^{a}	**3.2^{b}**	129.5^{b}	**144.2^{b}**	**174.7^{b}**	**1.1^{b}**	**4.4^{b}**	0.5^{a}
WD-1. ferment +5% red pomace	15.7^{ab}	**885.1^{b}**	184.9^{a}	0.0^{a}	0.7^{ab}	12.5^{ab}	9.1^{a}	5.2^{a}	1.7^{ab}	24.9^{ab}	34.8^{ab}	41.2^{ab}	0.5^{a}	0.8^{ab}	0.0^{a}
WD-1. ferment +10% red pomace	23.5^{ab}	**1348.8^{b}**	274.2^{a}	0.0^{a}	0.8^{ab}	14.8^{b}	11.5^{a}	4.0^{a}	1.8^{ab}	36.8^{ab}	49.6^{ab}	**59.5^{b}**	0.6^{a}	1.0^{ab}	0.3^{a}
WD-1. ferment +20% red pomace	72.9^{ab}	**4288.2^{b}**	**727.7^{b}**	0.0^{a}	**1.4^{b}**	**28.4^{b}**	24.2^{a}	6.2^{a}	**2.9^{b}**	107.1^{ab}	**127.2^{b}**	**152.1^{b}**	**1.1^{b}**	**4.0^{b}**	0.5^{a}
WD-1. ferment +30% red pomace	**82.8^{b}**	**4823.1^{b}**	**760.2^{b}**	0.0^{a}	**1.5^{b}**	**27.3^{b}**	23.2^{a}	5.4^{a}	**2.9^{b}**	119.6^{ab}	**118.6^{b}**	**147.0^{b}**	**1.1^{b}**	**4.2^{b}**	0.4^{a}
WD-2. ferment +5% red pomace	12.9^{ab}	**717.7^{b}**	154.8^{ab}	0.0^{a}	0.7^{a}	13.2^{ab}	8.8^{a}	5.2^{a}	1.6^{a}	20.9^{ab}	30.1^{ab}	35.9^{ab}	0.5^{ab}	0.6^{ab}	0.0^{a}
WD-2. ferment +10% red pomace	42.7^{ab}	**2629.3^{b}**	511.1^{ab}	0.0^{a}	**1.1^{b}**	**28.4^{b}**	22.0^{a}	8.5^{a}	**2.7^{b}**	73.1^{ab}	**91.3^{b}**	**110.8^{b}**	**0.8^{b}**	2.8^{ab}	0.5^{a}
WD-2. ferment +20% red pomace	61.0^{ab}	**3627.8^{b}**	**632.7^{b}**	0.0^{a}	**1.3^{b}**	**27.8^{b}**	21.3^{a}	6.0^{a}	**2.9^{b}**	91.8^{ab}	**103.6^{b}**	**127.3^{b}**	**0.9^{b}**	3.4^{ab}	0.4^{a}
WD-2. ferment +30% red pomace	**77.7^{b}**	**4592.9^{b}**	**725.3^{b}**	0.0^{a}	**1.4^{b}**	**27.8^{b}**	22.6^{a}	5.6^{a}	**2.9^{b}**	108.6^{ab}	**121.8^{b}**	**145.5^{b}**	**0.9^{b}**	3.9^{ab}	0.5^{a}
Wheat bun+5% red pomace	8.1^{ab}	**374.5^{b}**	84.3^{a}	0.0^{a}	0.8^{ab}	6.8^{ab}	6.5^{a}	3.3^{a}	1.6^{a}	21.0^{ab}	24.5^{ab}	27.4^{ab}	0.5^{ab}	0.4^{a}	0.0^{a}
Wheat bun+10% red pomace	13.5^{ab}	**726.5^{b}**	157.7^{a}	0.0^{a}	**0.9^{b}**	8.6^{ab}	9.5^{a}	3.3^{a}	1.9^{ab}	40.2^{ab}	45.4^{ab}	**51.8^{b}**	0.6^{ab}	1.0^{ab}	0.0^{a}
Wheat bun+20% red pomace	69.6^{ab}	**4207.7^{b}**	**745.7^{b}**	0.0^{a}	**2.4^{b}**	**34.2^{b}**	34.4^{a}	9.8^{a}	**4.9^{b}**	**210.3^{b}**	**223.8^{b}**	**243.8^{b}**	**1.7^{b}**	**6.8^{b}**	**0.5^{b}**
Wheat bun+30% red pomace	**74.4^{b}**	**4438.5^{b}**	**743.0^{b}**	0.0^{a}	**2.7^{b}**	**36.7^{b}**	35.6^{a}	8.7^{a}	**5.5^{b}**	**232.1^{b}**	**247.9^{b}**	**256.7^{b}**	**1.9^{b}**	**7.4^{b}**	**0.5^{b}**

Values not sharing any common letter indexes are considered significantly different at $p \leq 0.05$.
Red grape pomace: Saint Laurent and André varieties. [1]The highest standard deviation observed for each phenolic compound in tabeled samples.

Table S4. Average content of phenolic compounds (µg/g) measured during processing of control wheat dough and dough with a defined proportion of white grape pomace - RR (positive ionization, ESI+); Kruskal-Wallis test followed by Dunn's post-hoc test

Processing / PHE (µg/g)	Delphinidin-3-O-galactoside	Petunidin-3-O-glucoside	Malvidin-3-O-galactoside	Neochlorogenic acid	Chlorogenic acid	Vanillic acid	Syringic acid	Ferulic acid	Taxifolin	Miquelianin	Procyanidine B1+B3	Procyanidine B2	Procyanidine A2	Trans-resveratrol	Cis-resveratrol
Max. standard. deviation[1]	-	1.9	7.9	0.0	0.1	1.5	0.3	0.7	0.4	17.3	4.4	3.2	0.1	0.7	0.0
Wheat dough (WD)	0.0^{a}	0.0^{a}	6.1^{a}	0.0^{a}	0.0^{a}	3.8^{a}	2.6^{a}	5.3^{a}	0.0^{a}	1.8^{a}	0.0^{a}	0.0^{a}	0.0^{a}	0.0^{a}	0.0^{a}
WD-1. fermentation	0.0^{a}	0.0^{a}	2.0^{a}	0.0^{a}	0.0^{a}	0.0^{a}	2.1^{a}	6.1^{a}	0.0^{a}	1.4^{a}	0.0^{a}	0.0^{a}	0.0^{a}	0.0^{a}	0.0^{a}
WD-2. fermentation	0.0^{a}	0.0^{a}	1.9^{a}	0.0^{a}	0.0^{a}	0.0^{a}	2.0^{a}	5.9^{a}	0.0^{a}	1.4^{a}	0.0^{a}	0.0^{a}	0.0^{a}	0.0^{a}	0.0^{a}
Wheat bun	0.0^{a}	0.0^{a}	7.9^{a}	0.0^{a}	0.6^{a}	6.2^{a}	2.7^{a}	15.0^{a}	0.0^{a}	1.5^{a}	0.0^{a}	0.0^{a}	0.0^{a}	0.0^{a}	0.0^{a}
WD+5% RR	0.0^{a}	4.1^{ab}	2.5^{a}	0.0^{a}	0.6^{a}	0.0^{a}	1.9^{a}	3.7^{a}	1.4^{a}	30.8^{ab}	7.6^{ab}	5.2^{a}	0.0^{a}	0.5^{a}	0.0^{a}
WD+10% RR	0.0^{a}	6.7^{ab}	3.6^{a}	0.0^{a}	0.6^{a}	3.4^{a}	1.8^{a}	**2.6^{b}**	1.4^{a}	71.5^{ab}	15.6^{ab}	11.2^{ab}	0.0^{a}	1.6^{ab}	0.4^{ab}
WD+20% RR	0.0^{a}	12.4^{ab}	5.2^{a}	0.5^{a}	0.8^{ab}	3.6^{a}	2.1^{a}	2.8^{ab}	1.7^{ab}	137.6^{b}	27.9^{ab}	20.5^{ab}	0.5^{ab}	3.3^{ab}	0.5^{ab}
WD+30% RR	0.0^{a}	**31.4^{b}**	10.4^{a}	**0.7^{b}**	**1.1^{b}**	10.1^{ab}	2.6^{a}	4.2^{ab}	**2.3^{b}**	**251.3^{b}**	**67.6^{b}**	**47.8^{b}**	0.6^{ab}	**9.1^{b}**	**0.6^{b}**
WD-1. ferment.+5% RR	0.0^{a}	5.1^{ab}	2.9^{a}	0.0^{a}	0.6^{a}	0.0^{a}	1.9^{a}	3.1^{ab}	1.3^{a}	37.3^{ab}	9.4^{ab}	6.5^{ab}	0.0^{a}	0.9^{ab}	0.0^{a}
WD-1. ferment.+10% RR	0.0^{a}	5.6^{ab}	3.4^{a}	0.0^{a}	0.6^{a}	3.4^{a}	1.9^{a}	**2.6^{b}**	1.4^{a}	64.5^{ab}	14.2^{ab}	10.2^{ab}	0.0^{a}	1.6^{ab}	0.4^{ab}
WD-1. ferment.+20% RR	0.0^{a}	25.7^{ab}	9.5^{a}	0.7^{ab}	**1.0^{b}**	11.2^{ab}	3.0^{a}	6.9^{a}	**2.6^{b}**	**217.4^{b}**	**58.4^{b}**	42.1^{ab}	0.6^{ab}	**7.7^{b}**	**0.6^{b}**
WD-1. ferment.+30% RR	0.0^{a}	26.5^{ab}	9.4^{a}	0.7^{ab}	**1.0^{b}**	10.4^{ab}	2.5^{a}	4.5^{ab}	**2.3^{b}**	**227.1^{b}**	**58.4^{b}**	42.0^{ab}	0.6^{ab}	**7.7^{b}**	**0.6^{b}**
WD-2. ferment.+5% RR	0.0^{a}	4.3^{ab}	2.6^{a}	0.0^{a}	0.6^{a}	0.0^{a}	1.9^{a}	3.3^{ab}	1.4^{a}	32.4^{ab}	8.2^{ab}	5.6^{a}	0.0^{a}	0.7^{ab}	0.0^{a}
WD-2. ferment.+10% RR	0.0^{a}	5.1^{ab}	3.2^{a}	0.0^{a}	0.6^{a}	3.3^{a}	1.9^{a}	2.7^{ab}	1.5^{a}	58.2^{ab}	12.6^{ab}	9.1^{ab}	0.0^{a}	1.4^{ab}	0.4^{ab}
WD-2. ferment.+20% RR	0.0^{a}	21.2^{ab}	8.2^{a}	0.7^{ab}	**1.0^{b}**	10.3^{ab}	2.8^{a}	6.4^{a}	**2.5^{b}**	**184.9^{b}**	50.9^{ab}	35.8^{ab}	0.6^{ab}	**7.4^{b}**	**0.6^{b}**
WD-2. ferment.+30% RR	0.0^{a}	20.7^{ab}	7.8^{a}	0.6^{ab}	**0.9^{b}**	9.1^{ab}	2.4^{a}	4.2^{ab}	**2.3^{b}**	**189.3^{b}**	48.1^{ab}	34.0^{ab}	0.6^{ab}	**6.5^{b}**	**0.5^{b}**
Wheat bun+5% RR	0.0^{a}	7.2^{ab}	3.4^{a}	0.0^{a}	0.8^{ab}	6.5^{ab}	2.6^{a}	7.5^{a}	1.9^{ab}	74.9^{ab}	21.4^{ab}	13.0^{ab}	0.0^{a}	2.1^{ab}	0.5^{ab}
Wheat bun+10% RR	0.0^{a}	10.9^{ab}	4.7^{a}	0.0^{a}	**1.0^{b}**	7.7^{ab}	2.5^{a}	6.1^{a}	**2.2^{b}**	**157.9^{b}**	38.4^{ab}	24.5^{ab}	0.5^{ab}	**4.2^{b}**	**0.5^{b}**
Wheat bun+20% RR	0.0^{a}	17.2^{ab}	6.5^{a}	**0.8^{b}**	**1.4^{b}**	8.6^{ab}	2.6^{a}	6.1^{a}	**2.6^{b}**	**258.2^{b}**	**64.5^{b}**	38.7^{ab}	0.7^{ab}	**7.3^{b}**	**0.5^{b}**
Wheat bun+30% RR	0.0^{a}	19.2^{ab}	7.1^{a}	**0.9^{b}**	**1.3^{b}**	8.2^{ab}	2.5^{a}	5.0^{ab}	**2.8^{b}**	**314.5^{b}**	**77.1^{b}**	**44.9^{b}**	0.7^{ab}	**8.7^{b}**	**0.6^{b}**

Values not sharing any common letter indexes are considered significantly different at $p \leq 0.05$.
White grape pomace (RR): Rhine Riesling. [1]The highest standard deviation observed for each phenolic compound in tabeled samples.

Table S5. Average content of phenolic compounds (μg/g) measured during processing of control wheat dough and dough with a defined proportion of white grape pomace - RR+MM (positive ionization, ESI+); Kruskal-Wallis test followed by Dunn's post-hoc test

Processing / PHE (μg/g)	Delphinidin-3-O-galactoside	Petunidin-3-O-glucoside	Malvidin-3-O-galactoside	Neochlorogenic acid	Chlorogenic acid	Vanillic acid	Syringic acid	Ferulic acid	Taxifolin	Miquelianin	Procyanidine B1+B3	Procyanidine B2	Procyanidine A2	Trans-resveratrol	Cis-resveratrol
Max. standard. deviation[1]	-	1.3	7.9	0.0	0.1	2.5	0.3	0.7	0.7	18.8	7.0	4.8	0.1	1.4	0.0
Wheat dough (WD)	0.0^{a}	0.0^{a}	6.1^{a}	0.0^{a}	0.0^{a}	3.8^{a}	2.6^{a}	5.3^{a}	0.0^{a}	1.8^{a}	0.0^{a}	0.0^{a}	0.0^{a}	0.0^{a}	0.0^{a}
WD-1. fermentation	0.0^{a}	0.0^{a}	2.0^{a}	0.0^{a}	0.0^{a}	0.0^{a}	2.1^{a}	6.1^{a}	0.0^{a}	1.4^{a}	0.0^{a}	0.0^{a}	0.0^{a}	0.0^{a}	0.0^{a}
WD-2. fermentation	0.0^{a}	0.0^{a}	1.9^{a}	0.0^{a}	0.0^{a}	0.0^{a}	2.0^{a}	5.9^{a}	0.0^{a}	1.4^{a}	0.0^{a}	0.0^{a}	0.0^{a}	0.0^{a}	0.0^{a}
Wheat bun	0.0^{a}	0.0^{a}	7.9^{a}	0.0^{a}	0.6^{a}	6.2^{a}	2.7^{a}	15.0^{a}	0.0^{a}	1.5^{a}	0.0^{a}	0.0^{a}	0.0^{a}	0.0^{a}	0.0^{a}
WD+5% RR+MM	0.0^{a}	0.0^{a}	2.0^{a}	0.0^{a}	0.6^{a}	3.5^{a}	1.9^{a}	3.0^{ab}	1.3^{a}	18.7^{ab}	9.0^{ab}	6.4^{ab}	0.0^{a}	0.5^{a}	0.0^{a}
WD+10% RR+MM	0.0^{a}	4.4^{ab}	2.6^{a}	0.0^{a}	0.6^{a}	5.8^{ab}	2.0^{a}	3.8^{ab}	1.5^{a}	58.7^{ab}	24.6^{ab}	19.9^{ab}	0.0^{a}	2.4^{ab}	0.4^{ab}
WD+20% RR+MM	0.0^{a}	5.9^{ab}	3.3^{a}	0.5^{ab}	0.7^{a}	6.1^{ab}	2.0^{a}	3.1^{ab}	1.7^{ab}	106.2^{ab}	39.8^{ab}	33.1^{ab}	0.5^{ab}	4.1^{ab}	0.5^{ab}
WD+30% RR+MM	0.0^{a}	12.9^{ab}	4.9^{a}	0.7^{ab}	**1.0^{b}**	10.5^{ab}	2.4^{a}	4.4^{ab}	**2.7^{b}**	**224.5^{b}**	**76.5^{b}**	**64.7^{ab}**	**0.8^{b}**	**9.2^{b}**	**0.6^{b}**
WD-1. ferment.+5% RR+MM	0.0^{a}	0.0^{a}	2.4^{a}	0.0^{a}	0.6^{a}	6.8^{ab}	2.4^{a}	6.0^{a}	1.6^{ab}	32.6^{ab}	13.9^{ab}	10.8^{ab}	0.0^{a}	1.1^{ab}	0.4^{a}
WD-1. ferment.+10% RR+MM	0.0^{a}	0.0^{a}	2.5^{a}	0.0^{a}	0.6^{a}	5.7^{ab}	2.1^{a}	3.8^{ab}	1.6^{a}	49.5^{ab}	20.7^{ab}	15.7^{ab}	0.0^{a}	1.8^{ab}	0.5^{ab}
WD-1. ferment.+20% RR+MM	0.0^{a}	10.5^{ab}	4.4^{a}	0.6^{ab}	0.9^{ab}	12.7^{ab}	2.7^{a}	6.8^{a}	**2.5^{b}**	**191.9^{b}**	**68.7^{b}**	**57.2^{b}**	**0.7^{b}**	**8.3^{b}**	**0.6^{b}**
WD-1. ferment.+30% RR+MM	0.0^{a}	10.9^{ab}	4.4^{a}	0.6^{ab}	**0.9^{b}**	9.7^{ab}	2.3^{a}	4.3^{ab}	**2.3^{b}**	**194.5^{b}**	**64.4^{b}**	**53.8^{b}**	**0.7^{b}**	**8.4^{b}**	**0.5^{b}**
WD-2. ferment.+5% RR+MM	0.0^{a}	0.0^{a}	2.1^{a}	0.0^{a}	0.6^{a}	5.8^{ab}	2.2^{a}	5.8^{a}	1.5^{a}	26.1^{ab}	11.5^{ab}	8.6^{ab}	0.0^{a}	1.0^{ab}	0.4^{a}
WD-2. ferment.+10% RR+MM	0.0^{a}	0.0^{a}	2.3^{a}	0.0^{a}	0.6^{a}	5.1^{a}	2.0^{a}	3.5^{ab}	1.6^{a}	42.0^{ab}	17.5^{ab}	13.6^{ab}	0.0^{a}	1.5^{ab}	0.4^{ab}
WD-2. ferment.+20% RR+MM	0.0^{a}	7.1^{ab}	3.5^{a}	0.6^{ab}	0.8^{ab}	11.3^{ab}	2.5^{a}	6.3^{a}	**2.4^{b}**	**147.9^{b}**	51.0^{ab}	42.6^{ab}	0.6^{ab}	**6.0^{b}**	**0.5^{b}**
WD-2. ferment.+30% RR+MM	0.0^{a}	19.1^{ab}	6.8^{a}	**0.8^{b}**	**1.3^{b}**	**19.1^{b}**	3.3^{a}	8.5^{a}	**4.5^{b}**	**340.2^{b}**	**117.1^{b}**	**96.9^{b}**	**0.9^{b}**	**15.3^{b}**	**0.7^{b}**
Wheat bun+5% RR+MM	0.0^{a}	0.0^{a}	2.0^{a}	0.0^{a}	0.6^{a}	2.7^{a}	2.0^{a}	4.4^{ab}	1.6^{a}	35.3^{ab}	13.7^{ab}	9.7^{ab}	0.0^{a}	0.9^{ab}	0.0^{ab}
Wheat bun+10% RR+MM	0.0^{a}	0.0^{a}	2.3^{a}	0.0^{a}	0.7^{a}	4.0^{a}	2.0^{a}	3.6^{ab}	1.8^{ab}	73.4^{ab}	25.0^{ab}	18.0^{ab}	0.0^{a}	1.9^{ab}	**0.4^{b}**
Wheat bun+20% RR+MM	0.0^{a}	4.9^{ab}	2.8^{a}	0.6^{a}	0.9^{ab}	5.3^{a}	2.1^{a}	3.9^{ab}	2.2^{ab}	**158.1^{b}**	50.8^{ab}	35.8^{ab}	0.6^{ab}	**4.4^{b}**	**0.5^{b}**
Wheat bun+30% RR+MM	0.0^{a}	6.7^{ab}	3.4^{a}	0.7^{ab}	**1.0^{b}**	5.9^{ab}	2.1^{a}	3.6^{ab}	**2.3^{b}**	**216.4^{b}**	**62.7^{b}**	46.1^{b}	0.7^{ab}	**6.3^{b}**	**0.5^{b}**

Values not sharing any common letter indexes are considered significantly different at $p \leq 0.05$.
White grape pomace (RR+MM): Rhine Riesling (RR) and Moravian Muscat (MM). [1]The highest standard deviation observed for each phenolic compound in tabeled samples.

Table S6. Average content of phenolic compounds (µg/g) measured during processing of control wheat dough and dough with a defined proportion of red grape pomace (negative ionization, ESI–); Kruskal-Wallis test followed by Dunn's post-hoc test

Samples / Phenolic compound (µg/g)	Gallic acid	p-OH benzoic acid	Caffeic acid	p-coumaric acid	Astilbin	Hyperoside + Isoquercetin	Rutin	Trifolin	Myricetin	Quercitrin	Quercetin	Kaempferol	Catechin	Epicatechin	Gallocatechin	Epigallocatechin	Epigallocatechin gallate	Epicatechin gallate	Catechin gallate
Max. standard. deviation[1]	6.2	0.1	0.2	0.2	0.8	8.5	25.0	0.7	0.1	1.7	2.6	-	44.6	36.9	0.2	0.2	0.1	6.0	2.5
Wheat dough (WD)	0.0^{a}	0.5^{a}	0.0^{a}	0.0^{a}	0.0^{a}	0.0^{a}	0.4^{a}	0.0^{a}	0.0^{a}	0.0^{a}	0.0^{a}	0.0^{a}	0.0^{a}	0.0^{a}	0.0^{a}	0.0^{a}	0.0^{a}	0.0^{a}	0.0^{a}
WD-1. fermentation	0.0^{a}	0.5^{a}	0.0^{a}	0.0^{a}	0.0^{a}	0.0^{a}	0.3^{a}	0.0^{a}	0.0^{a}	0.0^{a}	0.0^{a}	0.0^{a}	0.0^{a}	0.0^{a}	0.0^{a}	0.0^{a}	0.0^{a}	0.0^{a}	0.0^{a}
WD-2. fermentation	0.0^{a}	0.6^{a}	0.0^{a}	0.0^{a}	0.0^{a}	0.0^{a}	0.1^{a}	0.0^{a}	0.0^{a}	0.0^{a}	0.0^{a}	0.0^{a}	0.0^{a}	0.0^{a}	0.0^{a}	0.0^{a}	0.0^{a}	0.0^{a}	0.0^{a}
Wheat bun	0.0^{a}	0.5^{a}	0.0^{a}	0.0^{a}	0.0^{a}	0.0^{a}	0.1^{a}	0.0^{a}	0.0^{a}	0.0^{a}	0.0^{a}	0.0^{a}	0.0^{a}	0.0^{a}	0.0^{a}	0.0^{a}	0.0^{a}	0.0^{a}	0.0^{a}
WD+5% red pomace	10.7^{a}	0.8^{a}	0.0^{a}	0.4^{a}	1.5^{a}	15.6^{a}	1.6^{a}	0.3^{a}	0.0^{a}	3.6^{a}	0.0^{a}	0.0^{a}	**253.7^{b}**	**222.0^{b}**	0.0^{a}	0.0^{a}	1.2^{a}	13.5^{a}	**4.6^{b}**
WD+10% red pomace	**21.4^{b}**	1.0^{a}	0.1^{a}	0.6^{a}	2.4^{a}	22.9^{a}	2.4^{a}	0.2^{a}	0.0^{a}	4.9^{a}	0.9^{a}	0.0^{a}	**396.8^{b}**	**350.3^{b}**	0.0^{a}	0.0^{a}	1.3^{a}	**25.3^{b}**	**8.3^{b}**
WD+20% red pomace	**45.6^{b}**	**1.2^{b}**	0.4^{a}	**0.9^{b}**	**4.4^{b}**	45.1^{a}	4.3^{a}	2.3^{a}	0.0^{a}	9.9^{a}	6.1^{a}	0.0^{a}	**608.0^{b}**	**546.1^{b}**	0.0^{a}	0.0^{a}	1.4^{a}	**50.6^{b}**	**17.1^{b}**
WD+30% red pomace	**67.5^{b}**	**1.5^{b}**	0.6^{a}	**1.1^{b}**	**6.4^{b}**	**65.2^{b}**	6.4^{a}	4.2^{a}	0.0^{a}	**14.4^{b}**	**12.4^{b}**	0.0^{a}	**723.9^{b}**	**656.5^{b}**	0.2^{a}	0.2^{a}	**1.6^{b}**	**70.1^{b}**	**24.6^{b}**
WD-1. ferment.+5% red pomace	11.1^{ab}	0.8^{ab}	0.1^{a}	0.8^{ab}	1.4^{ab}	10.0^{ab}	1.3^{a}	0.0^{a}	0.0^{a}	1.8^{ab}	0.1^{a}	0.0^{a}	**257.1^{b}**	**223.4^{b}**	0.0^{a}	0.0^{a}	1.2^{a}	12.9^{ab}	4.3^{ab}
WD-1. ferment.+10% red pomace	23.3^{b}	1.0^{ab}	0.3^{a}	**1.0^{b}**	2.3^{ab}	21.8^{ab}	2.3^{a}	0.2^{a}	0.0^{a}	4.5^{ab}	2.0^{ab}	0.0^{a}	**388.2^{b}**	**344.4^{b}**	0.0^{a}	0.0^{a}	1.3^{ab}	**25.5^{b}**	**8.3^{b}**
WD-1. ferment.+20% red pomace	52.3^{b}	**1.3^{b}**	0.5^{a}	**1.1^{b}**	**4.4^{b}**	45.0^{ab}	4.4^{a}	2.3^{a}	0.0^{a}	9.8^{ab}	7.4^{ab}	0.0^{a}	**618.6^{b}**	**547.7^{b}**	0.0^{a}	0.0^{a}	1.4^{ab}	**50.4^{b}**	**16.6^{b}**
WD-1. ferment.+30% red pomace	71.1^{b}	**1.6^{b}**	**0.7^{b}**	**1.3^{b}**	**6.1^{b}**	**63.3^{b}**	6.1^{a}	4.1^{a}	0.0^{a}	**14.0^{b}**	**14.7^{b}**	0.0^{a}	**721.1^{b}**	**643.7^{b}**	0.3^{a}	0.3^{a}	**1.6^{b}**	**68.6^{b}**	**24.2^{b}**
WD-2. ferment.+5% red pomace	11.1^{ab}	0.8^{ab}	0.2^{ab}	**1.1^{b}**	1.3^{a}	8.4^{ab}	1.2^{a}	0.0^{a}	0.0^{a}	1.4^{ab}	0.6^{ab}	0.0^{a}	**243.2^{b}**	**211.6^{b}**	0.0^{a}	0.0^{a}	1.2^{a}	12.2^{ab}	4.0^{ab}
WD-2. ferment.+10% red pomace	**21.8^{b}**	0.9^{ab}	0.3^{ab}	**1.1^{b}**	2.2^{ab}	20.0^{ab}	2.2^{a}	0.1^{a}	0.0^{a}	3.9^{ab}	2.0^{ab}	0.0^{a}	**362.7^{b}**	**320.7^{b}**	0.0^{a}	0.0^{a}	1.3^{ab}	**23.5^{b}**	**7.7^{b}**
WD-2. ferment.+20% red pomace	**54.7^{b}**	**1.3^{b}**	0.6^{ab}	**1.4^{b}**	**4.4^{b}**	44.7^{ab}	4.4^{a}	2.4^{a}	0.0^{a}	9.6^{ab}	7.0^{ab}	0.0^{a}	**602.6^{b}**	**535.3^{b}**	0.0^{a}	0.0^{a}	1.4^{ab}	**48.9^{b}**	**16.0^{b}**
WD-2. ferment.+30% red pomace	**74.7^{b}**	**1.6^{b}**	**0.9^{b}**	**1.5^{b}**	**6.2^{b}**	**64.0^{b}**	6.2^{a}	4.2^{a}	0.0^{a}	**14.5^{b}**	**13.2^{b}**	0.0^{a}	**716.2^{b}**	**643.2^{b}**	0.4^{a}	0.4^{a}	**1.6^{b}**	**66.4^{b}**	**23.2^{b}**
Wheat bun+5% red pomace	16.6^{ab}	0.7^{ab}	0.4^{ab}	**1.1^{b}**	1.3^{ab}	9.5^{ab}	1.4^{a}	0.0^{a}	0.0^{a}	1.4^{ab}	6.4^{ab}	0.0^{a}	220.2^{ab}	**186.3^{b}**	0.0^{a}	0.0^{a}	1.3^{ab}	14.9^{ab}	**5.1^{b}**
Wheat bun+10% red pomace	**30.0^{b}**	0.9^{ab}	**0.7^{b}**	**1.3^{b}**	2.4^{ab}	21.9^{ab}	2.4^{a}	0.1^{a}	0.0^{a}	4.1^{ab}	**10.1^{b}**	0.0^{a}	**342.5^{b}**	**294.7^{b}**	0.0^{a}	0.0^{a}	1.4^{ab}	**29.2^{b}**	**9.6^{b}**
Wheat bun+20% red pomace	**41.7^{b}**	**1.2^{b}**	**0.9^{b}**	**1.4^{b}**	**4.7^{b}**	51.5^{ab}	5.1^{a}	2.5^{a}	0.0^{a}	9.8^{ab}	**13.3^{b}**	0.0^{a}	**487.9^{b}**	**434.7^{b}**	0.0^{a}	0.0^{a}	**1.6^{b}**	**52.8^{b}**	**18.0^{b}**
Wheat bun+30% red pomace	**65.7^{b}**	**1.5^{b}**	**1.5^{b}**	**1.7^{b}**	**6.8^{b}**	**74.6^{b}**	**19.1^{b}**	4.3^{a}	0.1^{a}	**14.7^{b}**	**25.5^{b}**	0.0^{a}	**646.0^{b}**	**567.1^{b}**	0.3^{a}	0.3^{a}	**1.8^{b}**	**74.0^{b}**	**25.7^{b}**

Values not sharing any common letter indexes are considered significantly different at $p \leq 0.05$.
pomace: Saint Laurent and André varieties. [1]The highest standard deviation observed for each phenolic compound in tabeled samples.

Table S7. Average content of phenolic compounds (µg/g) measured during processing of control wheat dough and dough with a defined proportion of white grape pomace - RR (negative ionization, ESI–); Kruskal-Wallis test followed by Dunn's post-hoc test

Samples / Phenolic compound (µg/g)	Gallic acid	p-OH benzoic acid	Caffeic acid	p-coumaric acid	Astilbin	Hyperoside + Isoquercetin	Rutin	Trifolin	Myricetin	Quercitrin	Quercetin	Kaempferol	Catechin	Epicatechin	Gallocatechin	Epigallocatechin	Epigallocatechin gallate	Epicatechin gallate	Catechin gallate
Max. standard. deviation[1]	2.2	0.2	0.1	0.1	0.2	7.6	5.1	1.2	-	2.4	2.9	1.3	13.0	12.6	0.2	-	0.0	1.5	0.3
Wheat dough (WD)	0.0^{a}	0.5^{a}	0.0^{a}	0.0^{a}	0.0^{a}	0.0^{a}	0.4^{a}	0.0^{a}	0.0^{a}	0.0^{a}	0.0^{a}	0.0^{a}	0.0^{a}	0.0^{a}	0.0^{a}	0.0^{a}	0.0^{a}	0.0^{a}	0.0^{a}
WD-1. fermentation	0.0^{a}	0.5^{a}	0.0^{a}	0.0^{a}	0.0^{a}	0.0^{a}	0.3^{a}	0.0^{a}	0.0^{a}	0.0^{a}	0.0^{a}	0.0^{a}	0.0^{a}	0.0^{a}	0.0^{a}	0.0^{a}	0.0^{a}	0.0^{a}	0.0^{a}
WD-2. fermentation	0.0^{a}	0.6^{a}	0.0^{a}	0.0^{a}	0.0^{a}	0.0^{a}	0.1^{a}	0.0^{a}	0.0^{a}	0.0^{a}	0.0^{a}	0.0^{a}	0.0^{a}	0.0^{a}	0.0^{a}	0.0^{a}	0.0^{a}	0.0^{a}	0.0^{a}
Wheat bun	0.0^{a}	0.5^{a}	0.0^{a}	0.0^{a}	0.0^{a}	0.0^{a}	0.1^{a}	0.0^{a}	0.0^{a}	0.0^{a}	0.0^{a}	0.0^{a}	0.0^{a}	0.0^{a}	0.0^{a}	0.0^{a}	0.0^{a}	0.0^{a}	0.0^{a}
WD+5% RR	2.9^{ab}	0.8^{ab}	0.2^{ab}	0.3^{ab}	1.0^{a}	6.0^{ab}	3.5^{ab}	2.8^{a}	0.0^{a}	0.7^{ab}	6.0^{ab}	0.0^{a}	51.9^{ab}	36.4^{a}	0.0^{a}	0.0^{a}	1.2^{a}	3.2^{a}	0.5^{ab}
WD+10% RR	**7.7^{ab}**	0.9^{ab}	0.4^{ab}	0.4^{ab}	1.9^{ab}	**58.1^{b}**	**6.9^{b}**	**7.6^{b}**	0.0^{a}	**18.5^{b}**	3.5^{ab}	0.0^{a}	98.7^{ab}	77.2^{ab}	0.0^{a}	0.0^{a}	1.3^{ab}	8.5^{ab}	1.6^{ab}
WD+20% RR	14.5^{ab}	**1.2^{b}**	**1.2^{b}**	**1.0^{b}**	**3.7^{b}**	**121.4^{b}**	**12.7^{b}**	**16.1^{b}**	0.0^{a}	**40.0^{b}**	**10.2^{b}**	0.0^{a}	167.3^{ab}	136.4^{ab}	0.0^{a}	0.0^{a}	**1.7^{b}**	18.7^{ab}	3.5^{ab}
WD+30% RR	19.6^{ab}	**1.1^{b}**	**0.7^{b}**	0.6^{ab}	**3.8^{b}**	**138.5^{b}**	**13.1^{b}**	**17.1^{b}**	0.0^{a}	**43.3^{b}**	8.0^{ab}	0.0^{a}	209.8^{ab}	172.4^{ab}	1.5^{a}	0.0^{a}	**1.7^{b}**	**21.7^{b}**	4.0^{ab}
WD-1. ferment. +5% RR	3.3^{ab}	0.7^{a}	0.1^{ab}	0.2^{ab}	1.1^{a}	26.5^{ab}	3.4^{ab}	2.8^{ab}	0.0^{a}	8.0^{ab}	0.5^{ab}	0.0^{a}	53.3^{ab}	39.4^{ab}	0.0^{a}	0.0^{a}	1.2^{ab}	3.5^{ab}	0.6^{ab}
WD-1. ferment. +10% RR	7.4^{ab}	0.9^{ab}	0.5^{ab}	0.5^{ab}	1.9^{ab}	47.2^{ab}	**6.6^{b}**	**7.3^{b}**	0.0^{a}	**14.0^{b}**	7.4^{ab}	0.0^{a}	91.8^{ab}	72.8^{ab}	0.0^{a}	0.0^{a}	1.3^{ab}	7.9^{ab}	1.4^{ab}
WD-1. ferment. +20% RR	10.1^{ab}	0.9^{ab}	0.4^{ab}	0.4^{ab}	2.4^{ab}	**77.5^{b}**	**8.2^{b}**	**9.8^{b}**	0.0^{a}	**22.1^{b}**	6.1^{ab}	0.0^{a}	130.9^{ab}	103.2^{ab}	0.0^{a}	0.0^{a}	**1.5^{b}**	11.6^{ab}	2.2^{ab}
WD-1. ferment. +30% RR	**24.2^{b}**	**1.2^{b}**	**1.0^{b}**	0.7^{ab}	**4.0^{b}**	**138.1^{b}**	**13.7^{b}**	**18.1^{b}**	0.0^{a}	**42.1^{b}**	**14.5^{b}**	0.0^{a}	220.9^{ab}	181.0^{ab}	1.8^{a}	0.0^{a}	**1.7^{b}**	**22.6^{b}**	4.2^{ab}
WD-2. ferment. +5% RR	3.3^{ab}	0.7^{a}	0.2^{ab}	0.3^{ab}	1.0^{a}	12.5^{ab}	3.4^{ab}	2.6^{ab}	0.0^{a}	2.7^{ab}	4.2^{ab}	0.0^{a}	49.7^{a}	35.7^{a}	0.0^{a}	0.0^{a}	1.2^{a}	3.3^{a}	0.5^{ab}
WD-2. ferment. +10% RR	6.4^{ab}	0.9^{ab}	0.5^{ab}	0.6^{ab}	1.8^{ab}	37.6^{ab}	6.4^{ab}	7.0^{ab}	0.0^{a}	10.1^{ab}	8.6^{ab}	0.0^{a}	90.0^{ab}	68.0^{ab}	0.0^{a}	0.0^{a}	1.3^{ab}	7.2^{ab}	1.3^{ab}
WD-2. ferment. +20% RR	13.0^{ab}	1.0^{ab}	0.6^{ab}	0.5^{ab}	2.5^{ab}	**76.1^{b}**	**8.7^{b}**	**10.7^{b}**	0.0^{a}	**21.6^{b}**	**11.2^{b}**	0.0^{a}	145.9^{ab}	116.9^{ab}	0.0^{a}	0.0^{a}	**1.5^{b}**	12.7^{ab}	2.4^{ab}
WD-2. ferment. +30% RR	**24.2^{b}**	**1.2^{b}**	**1.0^{b}**	**0.8^{b}**	**3.8^{b}**	**127.9^{b}**	**13.2^{b}**	**17.4^{b}**	0.0^{a}	**38.4^{b}**	**17.2^{b}**	0.7^{a}	213.0^{ab}	173.0^{ab}	1.6^{a}	0.0^{a}	**1.7^{b}**	20.9^{ab}	3.9^{ab}
Wheat bun + 5% RR	4.9^{ab}	0.6^{a}	0.2^{ab}	0.2^{ab}	0.9^{a}	5.0^{ab}	3.4^{ab}	2.0^{ab}	0.0^{a}	0.3^{ab}	**13.7^{b}**	0.0^{a}	53.1^{ab}	39.4^{ab}	0.0^{a}	0.0^{a}	1.2^{ab}	3.5^{ab}	0.7^{ab}
Wheat bun + 10% RR	12.2^{ab}	0.9^{ab}	**0.7^{b}**	**0.5^{ab}**	1.7^{ab}	37.8^{ab}	6.1^{ab}	6.5^{ab}	0.0^{a}	9.4^{ab}	**19.8^{b}**	0.0^{a}	101.6^{ab}	78.6^{ab}	0.0^{a}	0.0^{a}	1.4^{ab}	8.9^{ab}	1.7^{ab}
Wheat bun + 20% RR	**22.1^{b}**	1.0^{ab}	**1.4^{b}**	0.8^{ab}	**3.1^{b}**	**91.9^{b}**	**12.5^{b}**	**13.2^{b}**	0.0^{a}	**26.4^{b}**	**26.5^{b}**	1.9^{a}	164.0^{ab}	129.9^{ab}	0.9^{a}	0.0^{a}	**1.6^{b}**	17.4^{ab}	3.3^{ab}
Wheat bun + 30% RR	**28.8^{b}**	**1.2^{b}**	**1.7^{b}**	**0.9^{b}**	**4.1^{b}**	**138.5^{b}**	**18.2^{b}**	**18.4^{b}**	0.0^{a}	**41.8^{b}**	**32.0^{b}**	2.0^{a}	208.3^{ab}	165.0^{ab}	**2.3^{b}**	0.0^{a}	**1.8^{b}**	**24.6^{b}**	**4.6^{b}**

Values not sharing any common letter indexes are considered significantly different at $p \leq 0.05$.

White grape pomace (RR+MM): Rhine Riesling (RR) and Moravian Muscat (MM). [1]The highest standard deviation observed for each phenolic compound in tabeled samples.

Table S8. Average content of phenolic compounds (μg/g) measured during processing of control wheat dough and dough with a defined proportion of white grape pomace - RR+MM (negative ionization, ESI–); Kruskal-Wallis test followed by Dunn's post-hoc test

Samples / Phenolic compound (μg/g)	Gallic acid	p-OH benzoic acid	Caffeic acid	p-coumaric acid	Astilbin	Hyperoside + Isoquercetin	Rutin	Trifolin	Myricetin	Quercitrin	Quercetin	Kaempferol	Catechin	Epicatechin	Gallocatechin	Epigallocatechin	Epigallocatechin gallate	Epicatechin gallate	Catechin gallate
St. dev. range (min. - max.)	5.1	0.1	0.2	0.1	0.4	8.9	1.1	1.5	-	17.2	4.1	-	19.2	16.6	0.6	-	0.1	3.0	0.6
Wheat dough (WD)	0.0^{a}	0.5^{a}	0.0^{a}	0.0^{a}	0.0^{a}	0.0^{a}	0.4^{a}	0.0^{a}	0.0^{a}	0.0^{a}	0.0^{a}	0.0^{a}	0.0^{a}	0.0^{a}	0.0^{a}	0.0^{a}	0.0^{a}	0.0^{a}	0.0^{a}
WD-1. fermentation	0.0^{a}	0.5^{a}	0.0^{a}	0.0^{a}	0.0^{a}	0.0^{a}	0.3^{a}	0.0^{a}	0.0^{a}	0.0^{a}	0.0^{a}	0.0^{a}	0.0^{a}	0.0^{a}	0.0^{a}	0.0^{a}	0.0^{a}	0.0^{a}	0.0^{a}
WD-2. fermentation	0.0^{a}	0.6^{a}	0.0^{a}	0.0^{a}	0.0^{a}	0.0^{a}	0.1^{a}	0.0^{a}	0.0^{a}	0.0^{a}	0.0^{a}	0.0^{a}	0.0^{a}	0.0^{a}	0.0^{a}	0.0^{a}	0.0^{a}	0.0^{a}	0.0^{a}
Wheat bun	0.0^{a}	0.5^{a}	0.0^{a}	0.0^{a}	0.0^{a}	0.0^{a}	0.1^{a}	0.0^{a}	0.0^{a}	0.0^{a}	0.0^{a}	0.0^{a}	0.0^{a}	0.0^{a}	0.0^{a}	0.0^{a}	0.0^{a}	0.0^{a}	0.0^{a}
WD+5% RR+MM	3.4^{ab}	0.7^{a}	0.0^{a}	0.1^{ab}	0.9^{a}	16.0^{ab}	2.2^{a}	1.1^{ab}	0.0^{a}	10.8^{ab}	0.8^{a}	0.0^{a}	58.7^{ab}	43.1^{ab}	0.0^{a}	0.0^{a}	1.2^{a}	3.6^{ab}	0.6^{ab}
WD+10% RR+MM	6.9^{ab}	0.9^{ab}	0.2^{ab}	0.3^{ab}	1.6^{ab}	39.4^{ab}	4.7^{ab}	4.6^{ab}	0.0^{a}	12.0^{ab}	0.5^{a}	0.0^{a}	115.8^{ab}	93.2^{ab}	0.0^{a}	0.0^{a}	1.3^{ab}	8.9^{ab}	1.8^{ab}
WD+20% RR+MM	16.6^{ab}	**1.2^{b}**	0.5^{ab}	0.6^{ab}	**3.2^{b}**	**91.9^{b}**	**9.2^{b}**	**11.9^{b}**	0.0^{a}	**28.4^{b}**	3.8^{ab}	0.0^{a}	214.2^{ab}	179.2^{ab}	0.5^{ab}	0.0^{a}	**1.6^{b}**	**21.8^{b}**	4.3^{ab}
WD+30% RR+MM	**27.2^{b}**	**1.4^{b}**	**1.0^{b}**	**0.9^{b}**	**5.2^{b}**	**150.7^{b}**	**14.6^{b}**	**19.8^{b}**	0.0^{a}	**47.8^{b}**	9.0^{ab}	0.0^{a}	**303.0^{b}**	**256.1^{b}**	**2.1^{b}**	0.0^{a}	**1.9^{b}**	**37.7^{b}**	**7.4^{b}**
WD-1. ferment.+5% RR+MM	2.3^{ab}	0.7^{a}	0.0^{a}	0.1^{ab}	0.8^{a}	7.7^{ab}	2.2^{a}	0.7^{ab}	0.0^{a}	7.0^{ab}	1.4^{ab}	0.0^{a}	51.5^{ab}	38.4^{ab}	0.0^{a}	0.0^{a}	1.3^{ab}	3.0^{a}	0.5^{ab}
WD-1. ferment.+10% RR+MM	7.1^{ab}	0.9^{ab}	0.2^{ab}	0.4^{ab}	1.6^{ab}	30.9^{ab}	4.2^{ab}	4.6^{ab}	0.0^{a}	8.4^{ab}	2.8^{ab}	0.0^{a}	116.4^{ab}	92.5^{ab}	0.0^{a}	0.0^{a}	1.3^{ab}	8.8^{ab}	1.8^{ab}
WD-1. ferment.+20% RR+MM	14.9^{ab}	**1.1^{b}**	0.6^{ab}	0.7^{ab}	**3.1^{b}**	**82.5^{b}**	**8.8^{b}**	**11.2^{b}**	0.0^{a}	**24.2^{b}**	6.0^{ab}	0.0^{a}	190.2^{ab}	161.1^{ab}	0.0^{a}	0.0^{a}	**1.5^{b}**	19.5^{ab}	4.1^{ab}
WD-1. ferment.+30% RR+MM	**31.0^{b}**	**1.5^{b}**	**1.1^{b}**	**1.0^{b}**	**5.0^{b}**	**141.0^{b}**	**14.1^{b}**	**19.3^{b}**	0.0^{a}	**44.9^{b}**	**12.3^{b}**	0.0^{a}	**300.0^{b}**	**255.0^{b}**	**2.2^{b}**	0.0^{a}	**1.9^{b}**	**35.7^{b}**	**7.0^{b}**
WD-2. ferment.+5% RR+MM	2.2^{ab}	0.8^{ab}	0.0^{a}	0.2^{ab}	0.8^{a}	4.4^{a}	2.3^{a}	1.0^{ab}	0.0^{a}	5.4^{ab}	2.8^{ab}	0.0^{a}	53.1^{ab}	37.2^{ab}	0.0^{a}	0.0^{a}	1.2^{a}	3.0^{a}	0.5^{ab}
WD-2. ferment.+10% RR+MM	5.7^{ab}	0.9^{ab}	0.2^{ab}	0.4^{ab}	1.4^{ab}	24.3^{ab}	4.2^{ab}	4.2^{ab}	0.0^{a}	6.1^{ab}	3.4^{ab}	0.0^{a}	108.5^{ab}	84.3^{ab}	0.0^{a}	0.0^{a}	1.3^{ab}	7.9^{ab}	1.5^{ab}
WD-2. ferment.+20% RR+MM	16.9^{ab}	**1.2^{b}**	**0.8^{b}**	**0.8^{b}**	**3.0^{b}**	**75.6^{b}**	**8.7^{b}**	**11.1^{b}**	0.0^{a}	**21.8^{b}**	8.5^{ab}	0.0^{a}	198.4^{ab}	161.1^{ab}	0.0^{a}	0.0^{a}	**1.5^{b}**	19.1^{ab}	3.8^{ab}
WD-2. ferment.+30% RR+MM	**28.3^{b}**	**1.4^{b}**	**1.1^{b}**	**1.1^{b}**	**4.5^{b}**	**120.4^{b}**	**12.8^{b}**	**16.9^{b}**	0.0^{a}	**37.6^{b}**	**16.8^{b}**	0.0^{a}	**289.3^{b}**	**245.4^{b}**	1.6^{ab}	0.0^{a}	**1.8^{b}**	**31.3^{b}**	**6.4^{b}**
Wheat bun+5% RR+MM	6.6^{ab}	0.7^{a}	0.3^{ab}	0.2^{ab}	0.9^{a}	6.3^{ab}	2.9^{ab}	1.5^{ab}	0.0^{a}	5.3^{ab}	**12.1^{b}**	0.0^{a}	68.2^{ab}	52.3^{ab}	0.0^{a}	0.0^{a}	1.2^{ab}	4.7^{ab}	0.9^{ab}
Wheat bun+10% RR+MM	14.3^{ab}	0.8^{ab}	0.6^{ab}	0.5^{ab}	1.7^{ab}	29.9^{ab}	4.7^{ab}	5.0^{ab}	0.0^{a}	7.1^{ab}	**14.1^{b}**	0.0^{a}	125.3^{ab}	101.8^{ab}	0.0^{a}	0.0^{a}	1.4^{ab}	11.1^{ab}	2.3^{ab}
Wheat bun+20% RR+MM	**28.3^{b}**	1.1^{ab}	**1.4^{b}**	**0.9^{b}**	**3.3^{b}**	**85.5^{b}**	**9.7^{b}**	**12.0^{b}**	0.0^{a}	**24.2^{b}**	**23.9^{b}**	0.1^{a}	216.8^{ab}	175.3^{ab}	1.2^{ab}	0.0^{a}	**1.7^{b}**	**25.9^{b}**	**5.2^{b}**
Wheat bun+30% RR+MM	**34.7^{b}**	**1.20^{b}**	**1.7^{b}**	**1.0^{b}**	**4.7^{b}**	**125.6^{b}**	**13.2^{b}**	**17.0^{b}**	0.0^{a}	**39.1^{b}**	**27.8^{b}**	1.0^{a}	**266.0^{b}**	**218.7^{b}**	**2.3^{b}**	0.0^{a}	**1.9^{b}**	**35.5^{b}**	**7.1^{b}**

Values not sharing any common letter indexes are considered significantly different at $p \leq 0.05$.
White grape pomace (RR+MM): Rhine Riesling (RR) and Moravian Muscat (MM). [1]The highest standard deviation observed for each phenolic compound in tabeled samples.

Table S9. Comparison of theoretical (TPR) and equivalent (EPR) pomace ratios for other phenolic compounds in red grape pomace during dough mixing, fermentation and baking (negative ionization, ESI–)

Processing / Phenolic compounds TRP (%) : ERP (%)	EPR: p-OH benzoic acid (%)	EPR: Caffeic acid (%)	EPR: p-coumaric acid (%)	EPR: Astilbin (%)	EPR: Rutin (%)	EPR: Trifolin (%)	EPR: Myricetin (%)	EPR: Quercitrin (%)	EPR: Quercetin (%)	EPR: Kaempferol (%)	EPR: Epicatechin (%)	EPR: Gallocatechin (%)	EPR: Epigallocatechin (%)	EPR: Epigallocatechin gallate (%)	EPR: Epicatechin gallate (%)	EPR: Catechin gallate (%)
WD: TRP (5%)	49.0	0.0	30.9	10.4	10.2	2.6	0.0	248.2	0.0	0.0	18.4	0.0	0.0	49.4	16.4	8.8
WD: TRP (10%)	60.4	5.7	41.9	17.0	14.7	2.3	0.0	337.5	3.1	0.0	29.0	0.0	0.0	51.5	30.9	16.0
WD: TRP (20%)	75.7	32.2	63.2	31.3	27.1	23.0	0.0	680.6	20.3	0.0	45.3	0.0	0.0	58.5	61.7	33.1
WD: TRP (30%)	95.0	55.4	79.4	45.5	40.0	41.8	0.0	992.2	41.6	0.0	54.4	8.5	11.5	65.3	85.5	47.5
WD: 1. ferment. TRP (5%)	48.0	7.1	61.9	9.7	8.0	0.0	0.0	122.4	0.4	0.0	18.5	0.0	0.0	48.0	15.7	8.3
WD: 1. ferment. TRP (10%)	60.1	22.9	75.4	16.6	14.5	2.1	0.0	312.1	6.8	0.0	28.6	0.0	0.0	51.5	31.1	16.0
WD: 1. ferment. TRP (20%)	78.3	44.9	83.2	31.3	27.7	22.9	0.0	675.2	24.8	0.0	45.4	0.0	0.0	58.3	61.4	32.0
WD: 1. ferment. TRP (30%)	96.1	67.3	96.4	43.7	38.0	41.0	0.0	962.0	49.1	0.0	53.4	11.2	15.2	64.9	83.7	46.7
WD: 2. ferment. TRP (5%)	48.7	17.6	78.7	9.1	7.6	0.0	0.0	94.5	1.9	0.0	17.5	0.0	0.0	47.9	14.8	7.6
WD: 2. ferment. TRP (10%)	54.7	24.0	80.3	15.8	13.5	0.5	0.0	266.8	6.8	0.0	26.6	0.0	0.0	51.0	28.7	14.9
WD: 2. ferment. TRP (20%)	80.4	56.8	101.2	31.2	27.7	23.7	0.0	660.1	23.4	0.0	44.4	0.0	0.0	58.2	59.6	30.9
WD: 2. ferment. TRP (30%)	100.2	79.0	114.5	44.3	39.0	42.6	0.0	997.6	44.2	0.0	53.3	15.8	21.5	64.6	80.9	44.7
Wheat bun: TRP (5%)	45.4	36.0	82.1	9.6	8.7	0.0	0.0	95.7	21.5	0.0	15.5	0.0	0.0	51.1	18.1	9.8
Wheat bun: TRP (10%)	56.2	65.4	97.5	17.0	15.0	1.3	0.0	282.4	33.7	0.0	24.4	0.0	0.0	55.0	35.6	18.5
Wheat bun: TRP (20%)	74.7	85.8	99.9	33.4	31.8	24.6	0.0	675.4	44.5	0.0	36.1	0.0	0.0	63.5	64.5	34.8
Wheat bun: TRP (30%)	93.9	135.9	127.5	48.7	119.2	42.9	10.9	1007.2	85.4	0.0	47.0	12.4	16.9	73.7	90.3	49.7

Red grape pomace: Saint Laurent and André varieties

Table S10. Comparison of theoretical (TPR) and equivalent (EPR) pomace ratios for other phenolic compounds in white grape pomaces (RR, RR+MM) during dough mixing, fermentation and baking (negative ionization, ESI–)

Processing / Phenolic compounds TRP (%) : ERP (%)	EPR: p-OH benzoic acid (%)	EPR: Caffeic acid (%)	EPR: p-coumaric acid (%)	EPR: Astilbin (%)	EPR: Rutin (%)	EPR: Trifolin (%)	EPR: Myricetin (%)	EPR: Quercitrin (%)	EPR: Quercetin (%)	EPR: Kaempferol (%)	EPR: Epicatechin (%)	EPR: Gallocatechin (%)	EPR: Epigallocatechin (%)	EPR: Epigallocatechin gallate (%)	EPR: Epicatechin gallate (%)	EPR: Catechin gallate (%)
WD: TPR (5%)	54.7	7.5	20.1	7.7	7.2	3.9	0.0	729.8	9.0	0.0	6.9	0.0	0.0	34.3	6.9	3.3
WD: TPR (10%)	64.6	18.3	35.1	14.1	14.7	12.3	0.0	3353.3	6.2	0.0	14.8	0.0	0.0	37.4	17.8	10.7
WD: TPR (20%)	87.9	54.4	75.7	27.8	27.9	28.7	0.0	7610.1	22.5	0.0	27.2	1.3	0.0	46.6	41.2	24.2
WD: TPR (30%)	96.8	53.5	70.0	36.5	35.9	38.7	0.0	10646.0	27.9	0.0	36.8	9.7	0.0	52.1	59.3	34.5
WD: 1. ferment. TPR (5%)	51.2	3.0	13.5	7.6	7.0	3.4	0.0	882.3	1.5	0.0	6.8	0.0	0.0	35.6	6.8	3.4
WD: 1. ferment. TPR (10%)	64.7	22.1	43.4	13.7	13.7	12.0	0.0	2468.4	16.4	0.0	14.3	0.0	0.0	36.9	17.0	10.0
WD: 1. ferment. TPR (20%)	75.1	29.1	50.4	22.2	22.0	22.0	0.0	5528.4	20.0	0.0	22.7	0.0	0.0	43.7	31.1	19.1
WD: 1. ferment. TPR (30%)	102.4	61.8	80.9	36.3	35.9	39.0	0.0	10077.7	43.5	0.5	37.6	10.6	0.0	52.3	58.4	34.1
WD: 2. ferment. TPR (5%)	54.6	7.3	20.2	7.3	7.2	3.5	0.0	309.6	8.7	0.0	6.4	0.0	0.0	34.1	6.5	3.1
WD: 2. ferment. TPR (10%)	68.0	22.3	46.3	13.1	13.5	11.3	0.0	1805.1	19.2	0.0	13.2	0.0	0.0	36.7	15.3	8.7
WD: 2. ferment. TPR (20%)	81.8	41.6	63.6	22.6	22.4	22.7	0.0	5120.5	31.5	0.4	24.0	0.0	0.0	43.7	32.0	19.1
WD: 2. ferment. TPR (30%)	97.7	63.4	89.2	33.6	33.5	35.6	0.0	8752.7	56.0	10.3	36.0	8.6	0.0	49.8	52.4	31.4
Wheat bun: TPR (5%)	48.7	15.0	19.4	7.1	8.1	3.7	0.0	145.6	39.4	0.0	7.9	0.0	0.0	35.5	8.3	5.0
Wheat bun: TPR (10%)	62.3	42.0	41.9	13.5	13.8	11.8	0.0	1903.1	54.0	0.0	15.6	0.0	0.0	39.2	20.2	12.6
Wheat bun: TPR (20%)	78.5	85.7	76.0	25.7	28.3	26.0	0.0	5874.5	81.5	29.8	26.3	5.5	0.0	46.8	43.5	26.1
Wheat bun: TPR (30%)	88.0	104.3	87.1	35.4	39.9	36.6	0.0	9266.8	95.6	46.2	33.1	12.4	0.0	52.5	60.5	35.9

White grape pomaces (mean ERP values): Rhine Riesling (RR); Rhine Riesling (RR) and Moravian Muscat (RR+MM)

Table S11. Comparison of theoretical (TPR) and equivalent (EPR) pomace ratios for other phenolic compounds in red grape pomace during dough mixing, fermentation and baking (positive ionization, ESI+)

Processing / Phenolic compounds TRP (%) : ERP (%)	EPR: Delphinidin-3-O-galactoside (%)	EPR: Neochlorogenic acid (%)	EPR: Chlorogenic acid (%)	EPR: Vanillic acid (%)	EPR: Syringic acid (%)	EPR: Ferulic acid (%)	EPR: Taxifolin (%)	EPR: Procyanidine B1+B3 (%)	EPR: Procyanidine B2 (%)	EPR: Procyanidine A2 (%)	EPR: Trans-resveratrol (%)	EPR: Cis-resveratrol (%)
WD: TPR (5%)	4.2	0.0	14.9	17.5	10.7	89.2	13.8	6.2	6.8	15.5	6.9	43.1
WD: TPR (10%)	5.9	0.0	16.3	18.0	13.7	66.0	14.5	8.0	9.0	16.4	6.3	0.0
WD: TPR (20%)	19.4	0.0	31.1	38.1	32.7	116.8	26.0	20.5	23.7	31.0	24.1	64.6
WD: TPR (30%)	20.7	0.0	30.4	35.2	28.9	86.9	26.8	20.7	23.4	29.4	22.3	63.5
WD: 1. ferment. TPR (5%)	3.3	0.0	13.8	17.7	11.1	101.3	13.6	5.0	5.5	14.0	4.2	0.0
WD: 1. ferment. TPR (10%)	5.0	0.0	15.5	21.0	13.9	78.0	14.8	7.1	8.0	15.5	5.3	36.5
WD: 1. ferment. TPR (20%)	15.4	0.0	27.2	40.3	29.4	121.4	24.0	18.3	20.4	29.0	20.0	62.9
WD: 1. ferment. TPR (30%)	17.5	0.0	28.8	38.7	28.2	105.3	24.1	17.0	19.7	28.1	21.2	61.1
WD: 2. ferment. TPR (5%)	2.7	0.0	13.0	18.7	10.7	101.0	13.3	4.3	4.8	13.1	2.8	0.0
WD: 2. ferment. TPR (10%)	9.0	0.0	21.6	40.2	26.6	166.2	22.1	13.1	14.8	22.1	14.2	64.6
WD: 2. ferment. TPR (20%)	12.9	0.0	24.5	39.4	25.8	116.6	23.9	14.9	17.1	23.3	17.4	60.3
WD: 2. ferment. TPR (30%)	16.4	0.0	27.0	39.3	27.4	109.1	23.7	17.5	19.5	23.7	19.9	62.6
Wheat bun: TPR (5%)	1.7	0.0	14.5	9.6	7.9	65.0	13.2	3.5	3.7	12.6	2.2	0.0
Wheat bun: TPR (10%)	2.9	0.0	17.5	12.1	11.5	64.3	15.5	6.5	6.9	16.1	5.0	0.0
Wheat bun: TPR (20%)	14.7	0.0	46.2	48.4	41.7	191.4	40.3	32.1	32.7	45.2	34.6	72.6
Wheat bun: TPR (30%)	15.7	0.0	51.0	51.9	43.1	170.8	45.4	35.6	34.4	49.7	37.7	70.4

Red grape pomace: Saint Laurent and André varieties

Table S12. Comparison of theoretical (TPR) and equivalent (EPR) pomace ratios for other phenolic compounds in white grape pomace during dough mixing, fermentation, and baking (positive ionization, ESI+)

Processing / Phenolic compounds TRP (%) : ERP (%)	EPR: Delphinidin-3-O-galactoside (%)	EPR: Neochlorogenic acid (%)	EPR: Chlorogenic acid (%)	EPR: Vanillic acid (%)	EPR: Syringic acid (%)	EPR: Ferulic acid (%)	EPR: Taxifolin (%)	EPR: Procyanidine B1+B3 (%)	EPR: Procyanidine B2 (%)	EPR: Procyanidine A2 (%)	EPR: Trans-resveratrol (%)	EPR: Cis-resveratrol (%)
WD: TPR (5%)	0.0	7.5	0.0	23.1	16.0	60.2	109.1	27.2	3.2	0.0	1.8	0.0
WD: TPR (10%)	0.0	10.0	0.0	26.1	38.2	61.5	107.3	29.5	8.5	0.0	6.8	50.9
WD: TPR (20%)	0.0	13.1	33.1	30.1	40.7	65.1	99.1	34.0	14.7	37.3	12.4	56.7
WD: TPR (30%)	0.0	22.0	43.6	40.5	83.4	77.8	141.9	50.9	30.9	49.9	30.6	65.9
WD: 1. ferment. TPR (5%)	0.0	8.7	0.0	25.0	30.8	69.0	153.7	29.7	4.7	0.0	3.4	28.7
WD: 1. ferment. TPR (10%)	0.0	9.4	0.0	25.1	38.1	62.5	107.4	30.8	7.1	0.0	5.7	50.8
WD: 1. ferment. TPR (20%)	0.0	19.7	41.2	38.6	97.8	88.2	225.8	51.1	27.2	48.9	26.8	68.0
WD: 1. ferment. TPR (30%)	0.0	19.7	40.9	38.6	81.1	76.6	144.6	47.0	26.3	47.4	27.0	64.1
WD: 2. ferment. TPR (5%)	0.0	7.8	0.0	23.2	26.3	65.6	154.1	29.5	3.9	0.0	2.9	27.5
WD: 2. ferment. TPR (10%)	0.0	8.7	0.0	24.3	34.8	61.1	104.6	31.3	6.2	0.0	4.9	49.0
WD: 2. ferment. TPR (20%)	0.0	16.2	39.3	35.0	87.9	84.2	209.9	48.7	21.6	43.6	22.4	65.2
WD: 2. ferment. TPR (30%)	0.0	24.5	46.7	45.1	119.1	92.6	215.8	69.7	35.3	52.8	37.1	70.8
Wheat bun: TPR (5%)	0.0	8.2	0.0	28.3	35.4	71.4	191.8	34.5	6.4	0.0	4.9	24.5
Wheat bun: TPR (10%)	0.0	10.0	0.0	33.5	45.6	69.3	156.8	39.4	11.9	20.3	10.1	53.1
Wheat bun: TPR (20%)	0.0	13.0	43.8	44.7	54.6	73.1	160.8	47.1	20.7	45.7	19.4	58.3
Wheat bun: TPR (30%)	0.0	15.1	49.2	47.4	56.0	72.1	138.8	50.7	25.2	50.4	24.8	60.4

White grape pomaces (mean ERP values): Rhine Riesling (RR); Rhine Riesling (RR) and Moravian Muscat (RR+MM)

Table S13 Rheological parameters of wheat dough supplemented with white grape pomace Riesling Rhine (RR), Riesling Rhine + Moravian Muscat (RR+MM), and red grape pomace Saint Laurent + André (RED) – Mixolab I

**Process / Parameters**	Water absorption (%)	Protein part of the curve			Starch part of the curve		
		C1 (min)	Stability (min)	C2 (Nm)	C3 (Nm)	C4 (Nm)	C5 (Nm)
Wheat – dough (WD)	61.2	3.97	9.52	0.330	2.540	1.250	1.670
WD+RR 5%	60.7	1.82	10.17	0.300	2.600	1.470	1.920
WD+RR 10%	61.0	1.30	9.47	0.300	2.720	2.150	2.930
WD+RR 20%	63.0	0.97	11.20	0.370	3.280	3.080	3.960
WD+RR 30%	67.1	0.68	0.53	0.340	2.940	2.690	3.190
WD+RR-MM: 5%	60.5	1.43	9.82	0.310	2.530	1.490	1.870
WD+RR-MM: 10%	60.8	1.07	9.50	0.330	2.760	2.280	2.870
WD+RR-MM: 20%	63.8	0.68	2.20	0.410	3.170	2.940	3.830
WD+RR-MM: 30 %	66.9	0.67	2.92	0.320	2.820	2.570	3.230
WD+RED 5%	59.8	2.18	10.77	0.320	2.660	1.590	2.110
WD+RED 10%	62.1	0.93	2.90	0.350	2.820	2.210	3.200
WD+RED 20%	68.9	0.93	1.22	0.130	0.850	0.300	3.490
WD+RED 30%	75.4	0.85	3.23	0.190	0.870	0.380	2.390

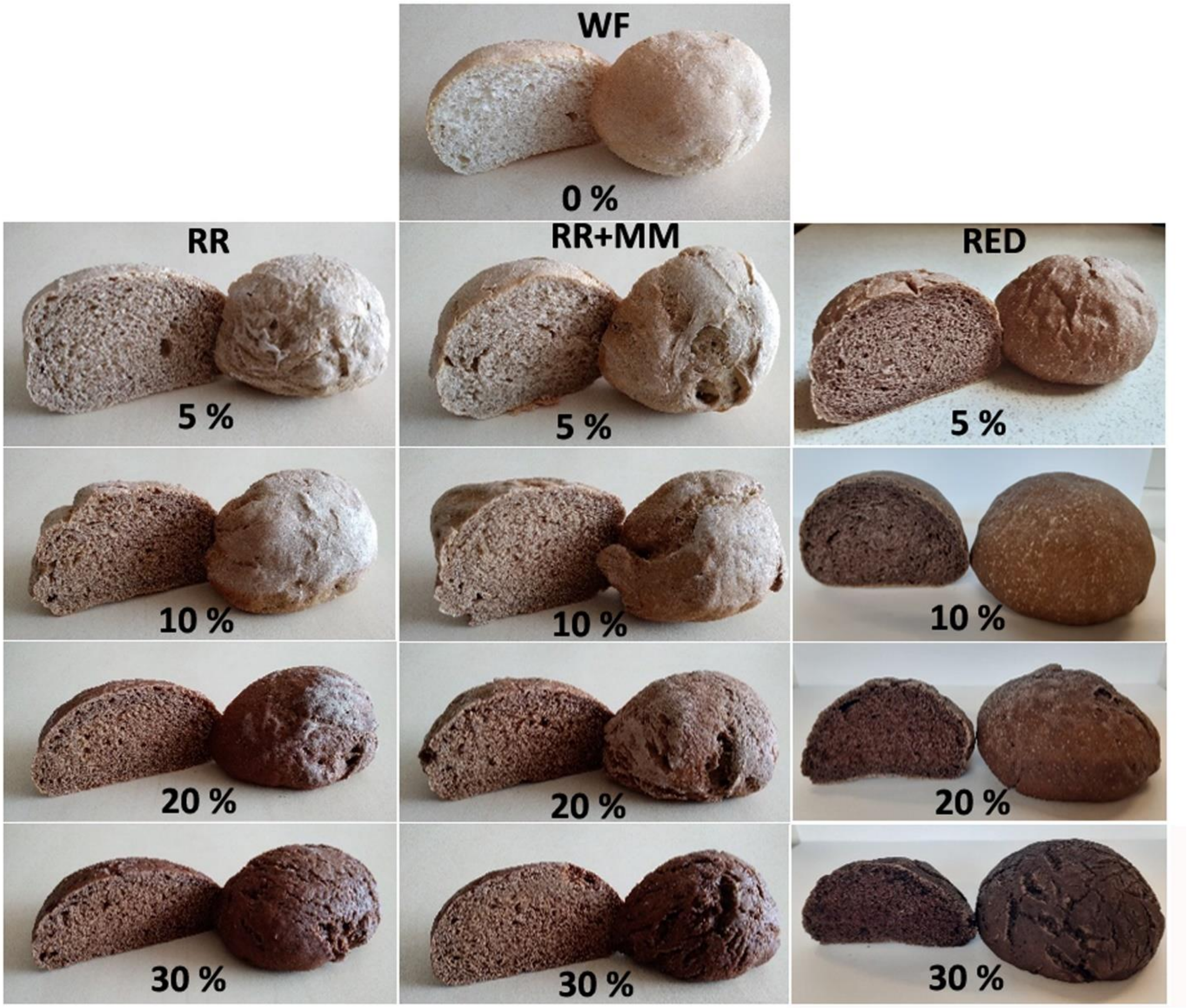

Figure S1 Wheat buns enriched with different proportions of white and red grape pomace

WF: Wheat flour
RED: Red grape pomace: Saint Laurent and André varieties; RR: White pomace - Rhine Riesling; RR+MM: White pomace - Rhine Riesling and Moravian Muscat
